# Supplementary material for: Wnt Signalosome Assembly by DEP Domain Swapping of Dishevelled
Source: Mol Cell. 2016 Oct 6;64(1):92–104. doi: 10.1016/j.molcel.2016.08.026 (PMC5065529; doi:10.1016/j.molcel.2016.08.026)
Supplement: Document S1. Supplemental Experimental Procedures, Figures S1–S7, and Tables S1 and S2 [file mmc1.pdf]

Molecular Cell, Volume *64*

Supplemental Information

Wnt Signalosome Assembly by DEP Domain Swapping of Dishevelled

Melissa V. Gammons, Miha Renko, Christopher M. Johnson, Trevor J. Rutherford, and Mariann Bienz

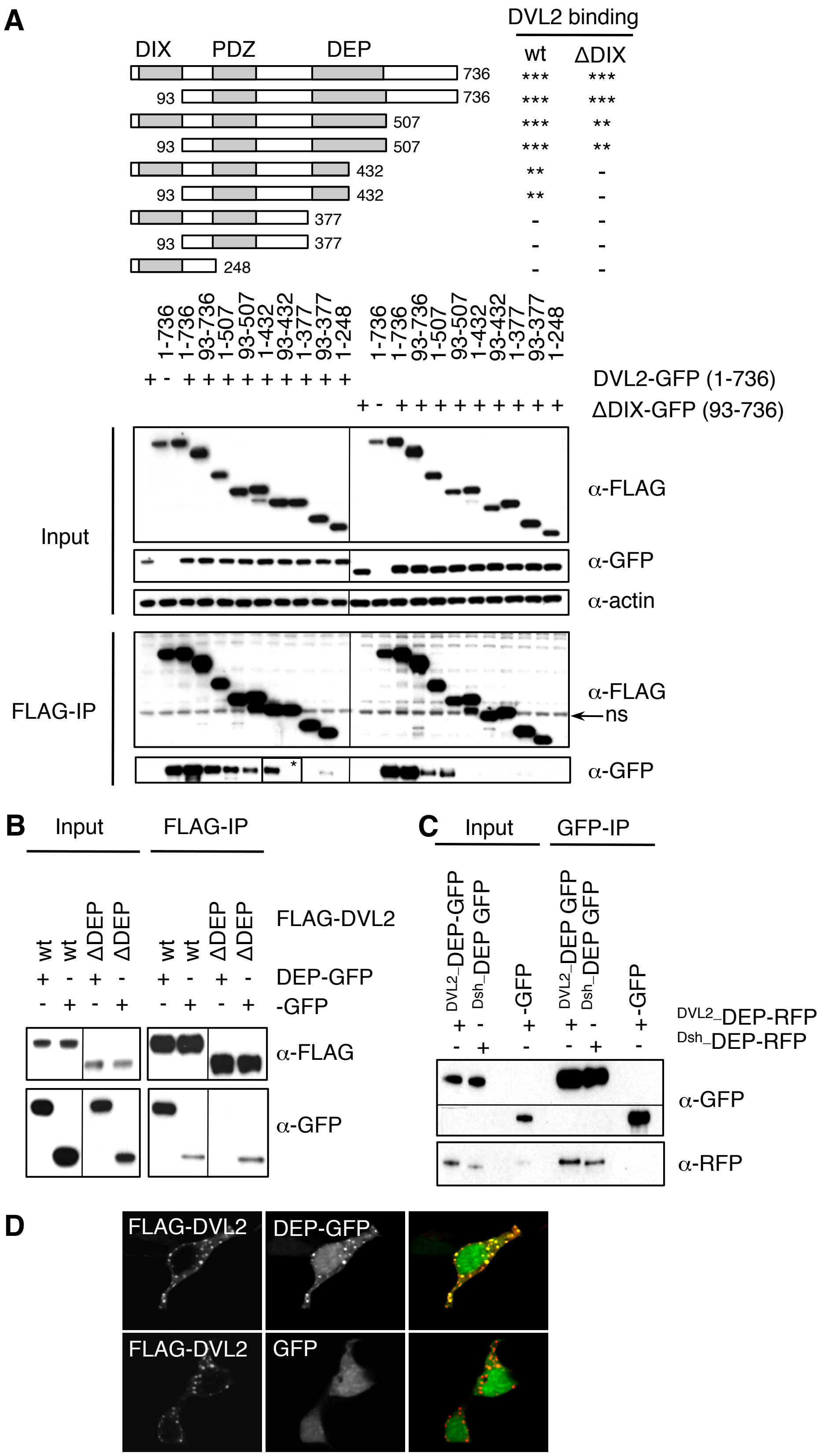

Fig. S1 (related to main Fig. 1)

DEP-dependent self-association of DVL2. (A-C) Western blots of coIPs from HEK293T cell lysates, 24 hours after transfection; (A) truncation mapping of DVL2 self-association reveals dependence on DEP, as summarized above; ns, non-specific band. (B-C) Mutual binding between DEP414-507-GFP and FLAG-DVL2 but not ΔDEP, or between DEP414-507-RFP and DEPDVL2-GFP or DEPDsh-GFP. (D) Confocal images of HEK293T cells co-expressing FLAG-DVL2 (red) and DEP414-507-GFP (green), revealing recruitment of DEP into DVL2 puncta; scale bar, 10µm. Lines indicate where blots have been spliced together, *lanes reversed due to misload.

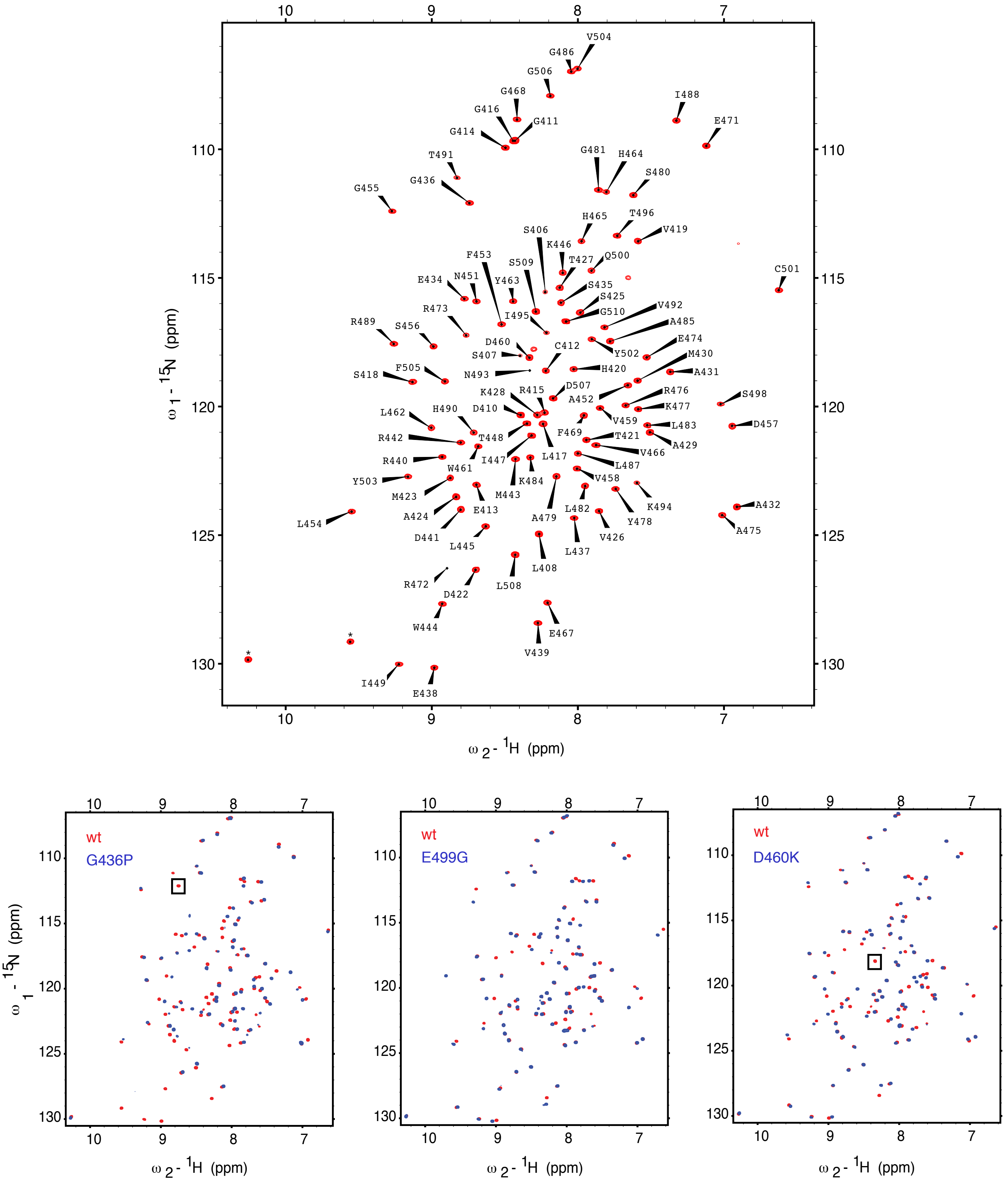

Fig. S2 (related to main Fig. 3)

BEST-TROSY spectra of wt and mutant DVL2 DEP. Top, 15N-1H correlation spectrum of purified 15N-13C-DEP416-511 monomer, with residues labelled according to backbone amide resonance assignments (except for A450, K470, N493, F497 and E499, which could not be assigned; asterisks indicate side chains); underneath, overlays of BEST-TROSY spectra of purified wt 15N-DEP416-511 (red) and mutants (blue) as indicated in panels, with mutated residues boxed (where assigned). The well-dispersed peaks of even intensity of the dimerization mutants indicate normal folding and absence of aggregation. The overall peak positioning for the wt spectrum is conserved in each of the mutants, and chemical shift mapping indicated that the only significant changes are in the vicinity of the mutated residue, although even these are relatively minor. We thus infer that the normal fold of the DEP monomer is preserved in each of the three mutants.

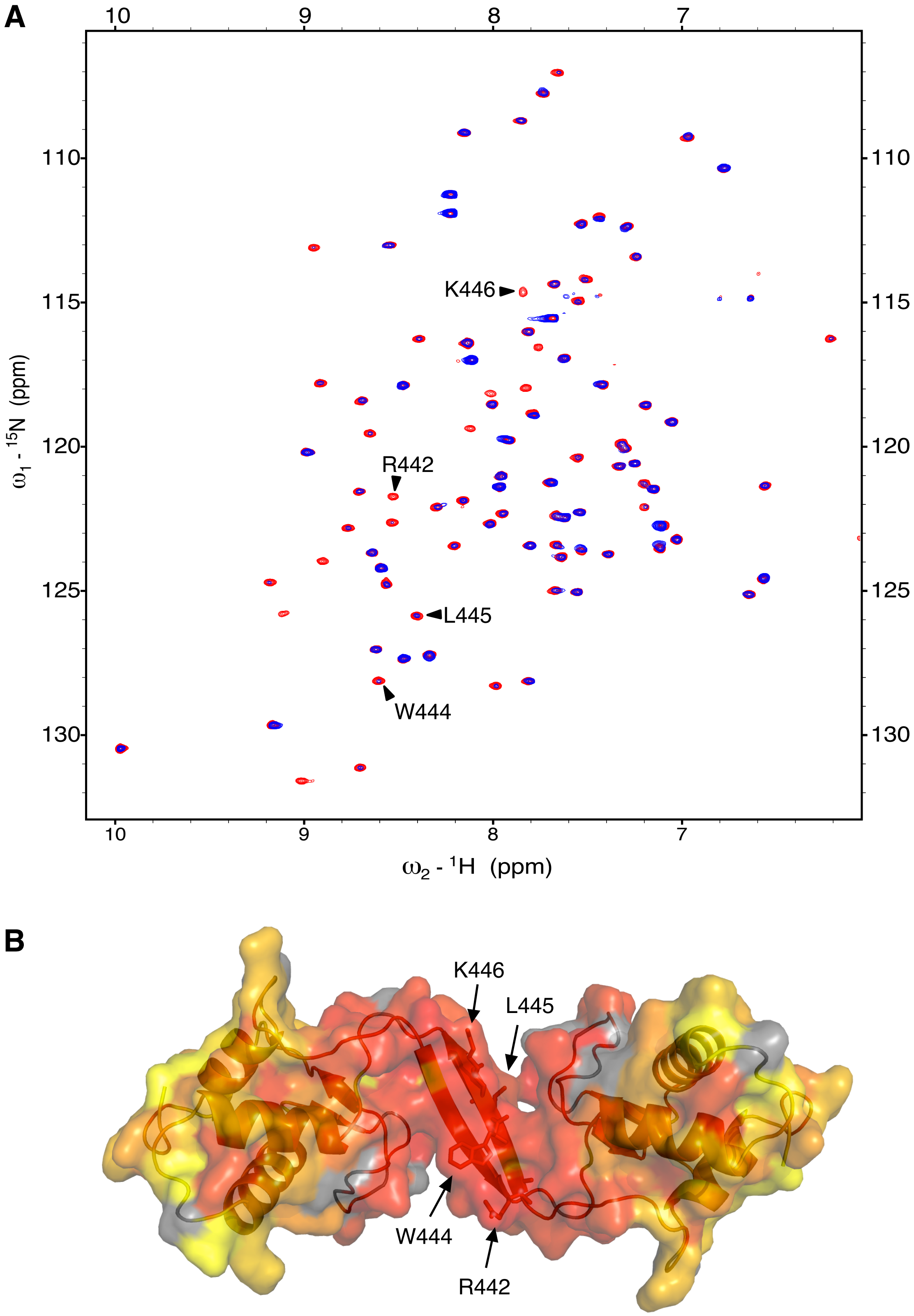

Fig. S3 (related to main Fig. 3)

BEST-TROSY spectra of monomeric and dimeric DEP. (A) Overlay of 15N-1H correlation spectra of 100 μM purified 15N-DEP416-511 monomer (red) and 15N-DEP416-511 dimer (blue); maximal line broadenings in the dimer (relative to the monomer) are observed for DEP finger residues (labeled), consistent with their radical conformational change from loop (in the monomer) to β1β2 sheet (in the dimer; see also main Fig. 2B). (B) Heat-map of relative line broadenings (dimer/monomer), ranging from 32.5% (yellow) to 77.4% reduction of absolute peak height (red), revealing maximal line broadenings in the β1β2 link region; grey, prolines and unassigned residues (see also Fig. S2).

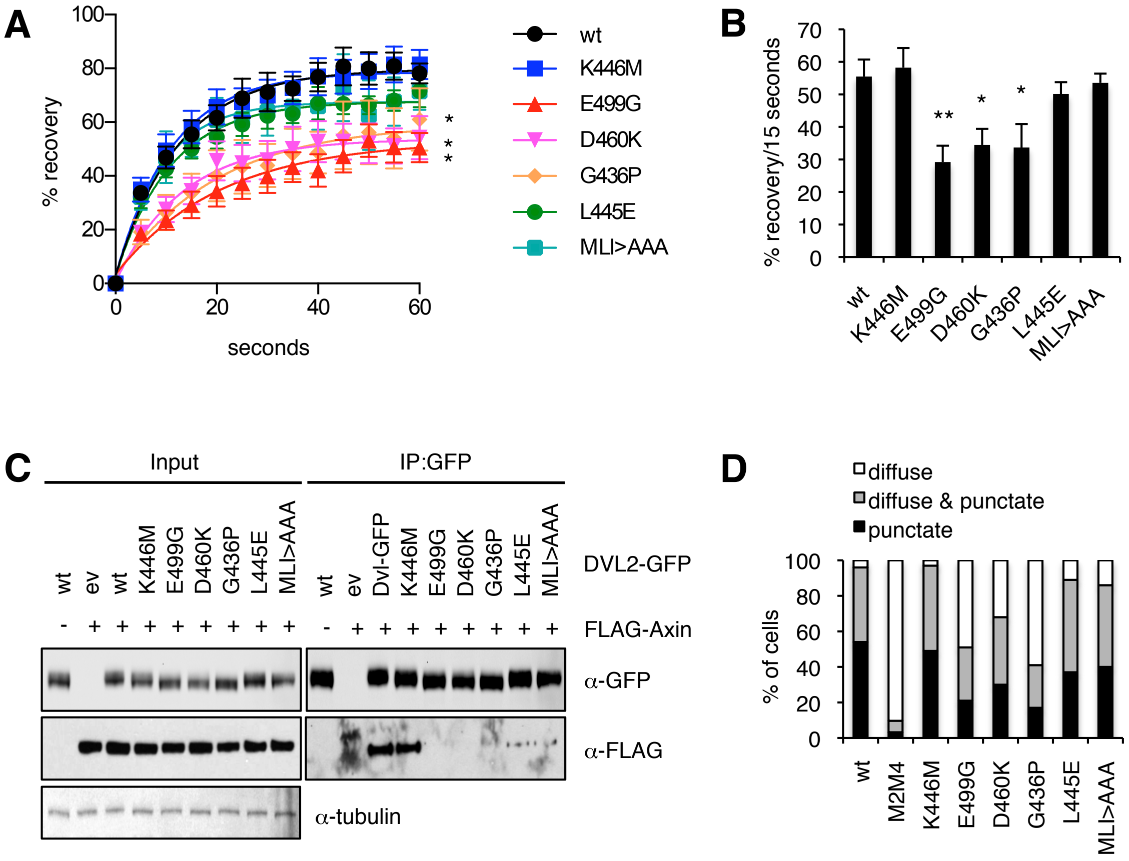

Fig. S4 (related to main Fig. 4)

Additional characterization of DEP mutants. (A, B) FRAP analysis of COS-7 cells expressing wt or mutant DVL2-GFP, as described (Metcalfe et al., 2010); >5 puncta were bleached per individual cell (>5 cells analyzed per mutant), and the fluorescence intensity was recorded every 5 seconds; (A) mean traces, with SEM indicated; (B) Percent recovery at 15 seconds; statistical significance, ** $p<0.01$, * $p<0.05$ (One-way ANOVA, Dunnetts post-hoc). (C) CoIP assays in transfected HEK293T cells, co-expressing DVL2-GFP and FLAG-Axin, as in main Fig. 1A. (D) Quantitation of puncta formation in HeLa cells expressing wt or mutant DVL2-GFP, as in main Fig. 2B (see also main Fig. 4D).

A

```
                    TM1                                ICL1        TM2

FZD5        229 ADERTFATFWIGLWSVLCFISTSTTVATFLI  DMER-FRYP   ERPIIFLSACYLCV-SLGFLVRL
Smo         224 EAEHQDMHSYIAAFGAVTGLCTLFTLATFVA  DWRNSNRYP   AVILFYVNACFFVG-SIGWLAQF
Rhodopsin    34 PWQFSMLAAYMFLLIVLGFPINFLTLYVTVQH   KKL-RT  PLNYILLNLAVADLFMVLGGFTSTLYTSLH
beta2AR      31  VWVVGMGIVMSLIVLAIVFGNVLVITAIAK   FERL-QT  VTNYFITSLACADLVMGLAVVPFGAAHILMK
A2AR          7     SVYITVELAIAVLAILGNVLVCWAVWL   NSNL-QN  VTNYFVVSLAAADIAVGVLAIPFAITISTGF

                ECL1                             TM3

FZD5        290 VVGHASVACSREHNHIHYETTGP            ALCTIVFLLVYFFGMASSIWWVILSLTWFLAAGMKW
Smo         264 MDGARREIVCRADGTMRLGEPTSNET         LSCVIIFVIVYYALMAGVVWFVVLTYAWHTSFKALG
Rhodopsin   101 GYFVFG                            PTGCNLEGFFATLGGEIALWSLVVLAIERYVVVC
beta2AR      98 MWTFG                             NFWCEFWTSIDVLCVTASIETLCVIAVDRYFAITS
A2AR         71 CAA                               CHGCLFIACFVLVLTQSSIFSLLAIAIDRYIAIR

                ICL2                   TM4                 ECL2

FZD5        349 GNE-AIAGYAQYF          HLAAWLIPSVKSITALAL SSVDGDPVAGICYVGNQN
Smo         348 TTYQPLSGKTSYF          HLLTWSLPFVLTVAILAV AQVDGDSVSGICFVGYKN
Rhodopsin   141    KPMSNF-RFG   ENHAIMGVAFTWVMALACAAPPLA  GWSRYIPEGLQCSCGIDYYTLKPEVN
beta2AR     138     PFKYQ-SLL  TKNKARVIILMVWIVSGLTSFLPIQM HWYRATHQEAINCYANETCCDFFT
A2AR        108    IPLRYN-GLVT  GTRAKGIIAICWVLSFAIGLTPML  GWNNCGQPKEGKNHSQGCGEGQVACLFEDVVP

                TM5                                       ICL3

FZD5        397 LNSLRGFVLGP-LVLYLLVGTLFLLAGFVSLFRIRSV     IKQGG-------------------TKT
Smo         397 YRYRAGFVLAP-IGLVLIVGGYFLIRGVMTLFSIKSN     HPGLLS-----------------EKAA
Rhodopsin   200 NESFVIYMFVVHFTIPMIIIFFCYGQLVFTVKEAAAQ     -QQ--------------------ES-A
beta2AR     196 NQAYAIASSIVSFYVPLVIMVFVYSRVFQEAKRQLQKIDKSE GRFHVQNLSQVEQDGRTGHGLRRSSKF
A2AR        174 MNYMVYFNFFACVLVPLLLMLGVYLRIFLAARRQLKQM     ESQPLPG------------ERARSTLQ

                TM6                                                       ECL3

FZD5        441 DKLEKLMIRIGIFTLLYTVPASIVVACYLYEQHYRESWEAALTCACPGHD TGQPRAKPE
Smo         443 SKINETMLRLGIFGFLAFGFVLITFSCHFYDFFNQAEWERSFRDYVLCQA NVTIGLPTKQPIPDCEIKNRPS
Rhodopsin   242 TTQKAEKEVTRMVIIMVIAFLICWVPYASVAFYIFTH              QGSNFGP
beta2AR     265   CLKEHKALKTLGIIMGTFTLCWLPFFIVNIVHVIQ              DNLI
A2AR        227     KEVHAAKSLAIIVGLFALCWLPLHIINCFTFF               CPDCSHAP

                TM7                             H8

FZD5        500      YWVLMLKYFMCLVVGITSGVWI  WS GKTVESWRRF
Smo         515      LLVEKINLFAMFGTGIAMSTWV  WT KATLLIWRRT
Rhodopsin   286   IFMTIPAFFAKS-AAIYNPVIYIMMN    KQFRNCMLTTIC
beta2AR     304 RKEVYILLNWIGYV-NSGFNPLIYCR    S PDFRIAFQEL
A2AR        267  LWLMYLAIVLSHT-NSVVNPFIYAYR  I  REFRQTFRKIIRSHVL
```

B

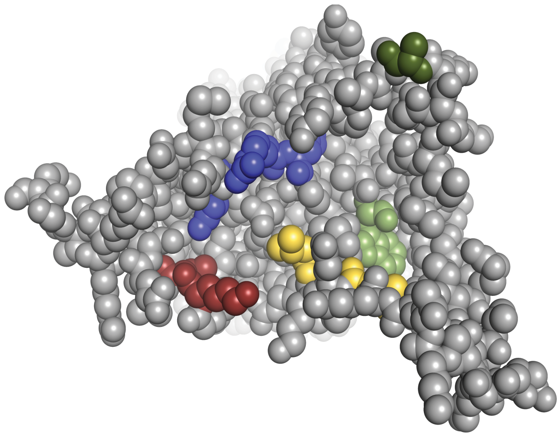

Fig. S5 (related to main Fig. 5)

Structural alignments of GPCRs. (A) Sequence alignments of human GPCRs mentioned in this study, based on structural projections generated by gpcrdb.org, but adopting the limits of TM segments (TM1-7) and α-helix 8 (H8) of Smoothened (Smo) and FZD5 from Fig. S1 (4JKV; Wang et al., 2013), of the adenosine A2A (A2AR) and β-adrenergic (beta2AR) receptors from Fig. S5 (Carpenter et al., 2016; see also 3SN6; Rasmussen et al., 2011), and of Rhodopsin from 4ZWJ (Kang et al., 2015). Sequence similarity was taken into account for alignments of intracellular loop sequences (ICL1-3) within and, where possible, across the two classes (although the cross-class alignments are arbitrary as these loops are largely unstructured, exhibiting little if any sequence conservation); extracellular segments (ECL1-3) were left-aligned, for simplicity. Blue, X50 residues in Ballesteros & Weinstein numbering as identified by gpcrdb.org, except for 2.50 whose topological equivalent in Smoothened is F274 (Y278 in FZD5), according to Fig. S1 (Wang et al., 2013) and our own structural cross-class alignments of TM2 segments. Residues required (red, orange) or dispensable (turquoise) for DEP recruitment to FZD5 are colored as in main Fig. 5C (red, expression level and modification comparable to wt FZD5; orange, abnormal expression, i.e. low level, or unmodified, or proteolyzed). Residues contacting Gs (green) or Arrestin (purple) were also colored; for Gs, see Fig. 3 & S5 (Carpenter et al., 2016); for Arrestin, see cross-linking data in Fig. 5 (Kang et al., 2015); ICL1 also contributes to Arrestin binding (Kang et al., 2015) although there are no cross-linking data for this loop. (B) Variant on-view of DEP binding to intracellular face of FZD5, as defined by residues required for DEP recruitment (red in A); rainbow colors as in main Fig. 5D. This model, based on the structure of Smoothened in complex with cyclopamine (4O9R; Weierstall et al., 2014), presents a somewhat more 'open' face compared to that shown in main Fig. 5D (based on 4JKV, the structure of Smoothened in complex with LY2940680; Wang et al., 2013), fully exposing W340 (light green), the topological equivalent of the arginine in DRY motif at the apex of the Gs binding pocket which, in class A GPCRs, engages in a crucial contact with the C-terminus of Gs (Carpenter et al., 2016; Rasmussen et al., 2011). Both models were generated by Phyre2.

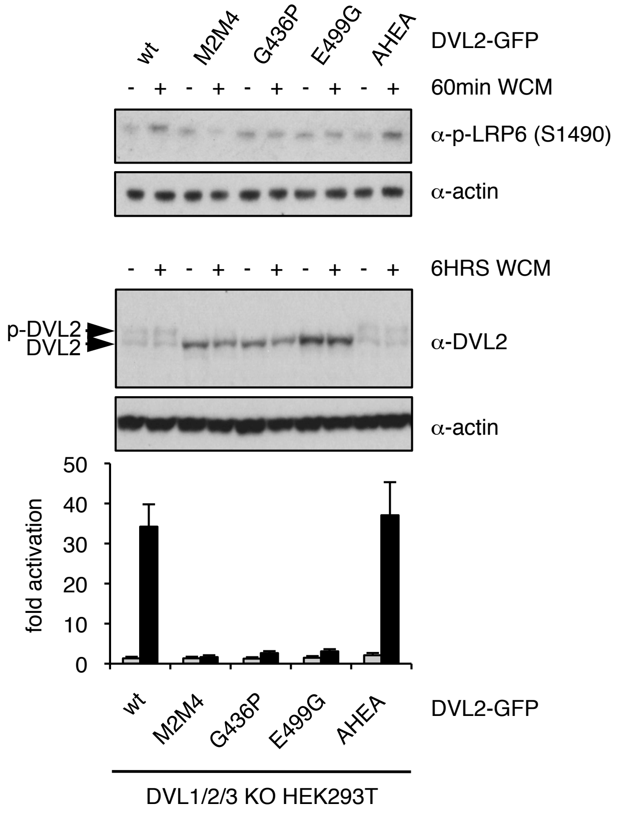

Fig. S6 (related to main Fig. 7)

Requirement for DEP-dependent dimerization during Wnt signaling. Complementation assays of wt or mutant DVL2-GFP expressed at physiological levels with a pBABEpuro retroviral vector in HEK293T cells lacking endogenous DVL1-3. (A) SuperTOP assays monitoring signaling activities of complementing transgenes (with corresponding Western blot above), as in main Fig. 1C. (B) GSK3-dependent phosphorylation of LRP6 (Zeng et al., 2005), as a more direct read-out of Dishevelled signaling activity (Bilic et al., 2007). Notably, these signaling activities of DVL2-GFP are strictly Wnt-dependent as they are detectable only after stimulation with Wnt3a-conditioned media (WCM, for 6 hours or 60 minutes, as indicated), but they do not depend on binding to AP2m since mutation of the m2-binding YHEL motif (located downstream of the DEP domain) to AHEA (Yu et al., 2010) has no detectable effect on function in these complementation assays.

Note: Our characterisation of the complementation assay is now in press (Gammons, M., Rutherford, T.J., Steinhart, Z., Angers, S., and Bienz, M. Essential role of the Dishevelled DEP domain in a Wnt-dependent human cell-based complementation assay. J. Cell Sci. 2016)

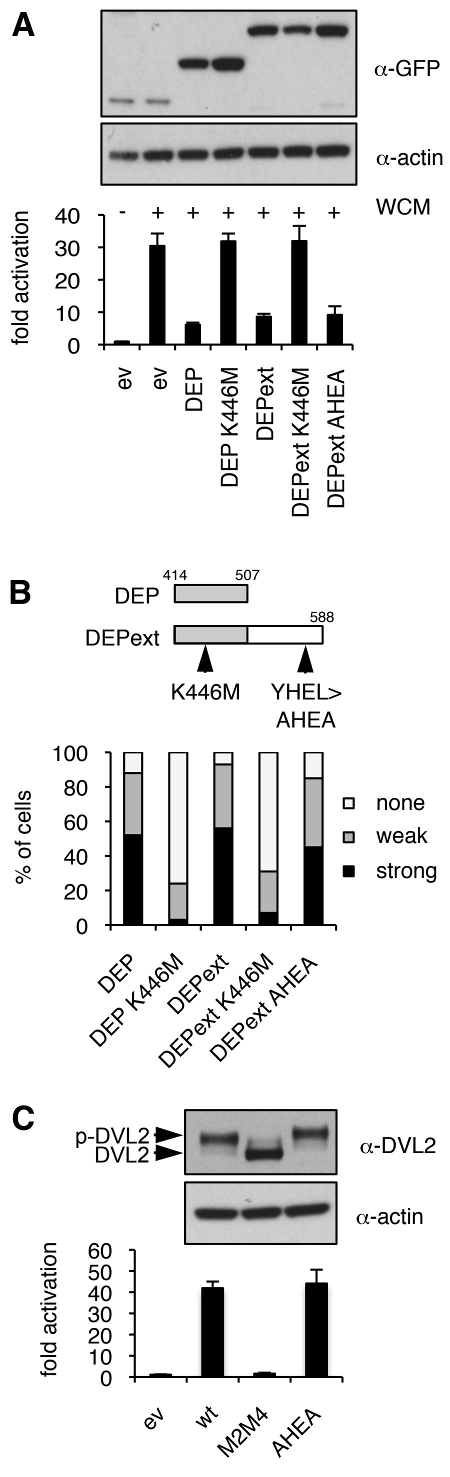

Fig. S7 (related to main Fig. 7)

Additional functional testing of the DVL2 YHEL motif. (A) SuperTOP assays in transiently transfected HEK293T cells, as in main Fig. 1C (with corresponding Western blot above). (B) SuperTOP assays, monitoring blocking of Wnt signal transduction by *endogenous* Dishevelled, as in main Fig. 6A (*above*, corresponding Western blot). (C) PM recruitments assays in HEK293T cells co-transfected with SNAP-FZD5 plus DEP-GFP, or plus an extended DEP domain including YHEL (DEPext-GFP; amino acids 414-588; Yu et al., 2010), as in main Fig. 6B. None of these assays uncovered a requirement for DVL2 binding to AP2m (blocked by the AHEA mutation; Yu et al., 2010) which appeared dispensable for Wnt-independent (A) and Wnt-dependent signaling (B), and also for recruitment to FZD5.

mutation	coIP	recruitment to DVL2 puncta	DVL2 signalling activity
S418A	Y	Y	+
T421A	Y	Y	+++
D422A	Y	Y	nt
S425A	Y	Y	+++
K428A	Y	reduced	nt
A432R	Y	Y	nt
E434R	Y	Y	nt
S435A	Y	Y	+
E438K	Y	Y	nt
R440H	Y	Y	nt
R442H	Y	Y	nt
R442W	Y	Y	nt
W444A	Y	Y	nt
K446M	Y	Y	++
K446E	Y	Y	++
T449R	Y	Y	nt
P450A	Y	Y	+++
E471A	Y	Y	nt
E471K	Y	Y	nt
R473E	Y	Y	nt
A475V	Y	Y	+
K477E	Y	Y	nt
Y478A	Y	Y	nt
S480A	Y	Y	++
I488N	Y	Y	+
T491R	Y	Y	nt
K494A	Y	Y	++
S498A	Y	Y	nt
Q500A	Y	Y	nt
C501R	Y	Y	+++
Y502F	Y	Y	+++

Table S1 (related to main Fig. 2)

Mutation screening of DEP surface residues. Effects of individual amino acid substitutions of DEP surface residues on DEP or DVL2 function in transfected HEK293T cells; first column, coIP of mutant DEP-GFP with wt DEP-RFP (as in Fig. S1C); second column, recruitment of mutant DEP-GFP to wt FLAG-Dvl2 puncta (as in Fig. S1D); third column, signaling activity of full-length Dvl2-GFP bearing DEP mutation; +++, wt; ++, >50% of wt; +, <50% of wt; nt, not tested.

PDB ID	5SUY	5LNP	5SUZ
Condition	0.1 M MES, pH=6.5<br>0.1 M NaCl<br>0.1 M Li_2SO_4<br>23 % PEG400	0.1 M MES, pH=6.5<br>0.1 M NaCl<br>0.1 M Li_2SO_4<br>23 % PEG400	0.1 M MES, pH=6.5<br>0.2 M $CaCl_2$<br>23 % PEG350 MME
Resolution range	40.5 - 1.89 (1.96 - 1.89)	42.0 - 1.99 (2.06 - 1.99)	32.7 - 1.84 (1.91 - 1.84)
Space group	$P2_1$	$P2_1$	C2
Unit cell	61.67, 60.17, 66.13,<br>90, 117.62, 90	61.58, 60.18, 66.29<br>90, 117.71, 90	117.79, 59.60, 30.32<br>90, 93.64, 90
Total reflections	236719 (3397)	200819	239437 (23533)
Unique reflections	34407 (3423)	29398 (2884)	17446 (1733)
Multiplicity	6.9 (6.9)	6.8 (6.6)	13.7 (13.6)
Complicity (%)	99.2 (98.3)	99.0 (98.6)	92.0 (96.0)
Mean I/sigma(I)	13.1 (1.8)	17.3 (2.5)	16.1 (0.98)
R-merge	0.085 (0.963)	0.064 (0.636)	0.107 (2.416)
R-meas	0.093 (1.042)	0.070 (0.690)	0.111 (2.511)
CC1/2	0.999 (0.767)	0.999 (0.889)	1 (0.937)
Twin fraction (%)	20.2	46.7	n/a
Twin operator	-h, -k, h+l	-h, -k, h+l	n/a
Reflections used in refinement	34407 (3423)	29398 (2884)	16251 (1215)
Reflections used for R-free	1837 (191)	1559 (150)	852 (79)
R-work	0.185	0.194	0.217
R-free	0.217	0.246	0.263
Number of non-hydrogen atoms:	3147	3031	1601
-macromolecules	3015	2993	1519
-ligands	15	5	n/a
Protein residues	378	377	190
RMS (bonds)	0.016	0.016	0.012
RMS (angles)	1.59	1.75	1.57
Ramachandran favored (%)	98	96	99
Ramachandran allowed (%)	2.1	3	0.53
Ramachandran outliers (%)	0	0.54	0.53
Rotomer outliers (%)	0.31	5.1	4.9
Clashscore	1.16	2.69	1.98
Average B-factor	33.05	37.09	39.83
-macromolecules	32.87	37.12	39.71
-ligands	55.20	47.43	n/a
-solvent	34.86	32.18	41.94

Statistics for the highest-resolution shell are shown in parentheses.

Table S2 (related to main Fig. 2)

Crystallography data collection and refinement statistics

SUPPLEMENTAL EXPERIMENTAL PROCEDURES

X-ray structure determination and refinement

Crystallization was done with concentrated DEP protein (20 mg/ml) after removal of tag by TEV protease (leaving two serine residues at the N-terminal end of DEP), as described (Stock et al., 2005); the initial screen involved ~1500 different crystallization conditions in 100 nl drops in a 96-well sitting-drop format. Crystals emerged under multiple conditions after growing for 30 days at 19ºC by the vapor diffusion method, and were directly flash-frozen in liquid nitrogen. X-ray diffraction data were collected at 100 K with a Dectris Pilatus 6M detector using the Diamond Light Source beamlines I04 and I04-1, from crystals grown in 0.1 M MES, pH 6.5, 0.2 M $CaCl_2$, 23 % PEG350 MME (C2 crystal form) or 0.1 M MES, pH 6.5, 0.1 M NaCl, 0.1 M Li_2SO4 and 22 % PEG400 ($P2_1$ crystal form). The C-centered monoclinic structure was determined by single anomalous dispersion using selenomethionine-labelled crystals. Determination of the heavy atom substructure and initial CA-model building were done using SHELXD (Sheldrick, 2010). The structure was built with ArpWarp (Langer et al., 2013) and manually edited subsequently with COOT (Emsley et al., 2010). The primitive monoclinic crystals were merohedrally twinned, with variable twinning fraction (20-45%). These structures were determined by molecular replacement with Phaser (McCoy et al., 2007), included in the CCP4 suite (Winn et al., 2011), using the C-centered monoclinic structure as a search model. All structures were refined using Refmac (Murshudov et al., 2011), and structural images were drawn with PyMol.

NMR spectroscopy

NMR spectra were acquired on Bruker Avance-III spectrometers operating at 600 or 800 MHz ^{1}H frequency, and equipped with cryogenic inverse 5 mm probes. Backbone resonance frequencies were obtained for 350 µM ^{13}C-^{15}N-labelled protein at 298 K, using unmodified Bruker pulse programs for HNCACB, CBCA(CO)NH, HN(CA)CO and HNCO. {^{1}H,^{15}N}-BEST-TROSY spectra (Favier and Brutscher, 2011) were acquired with 128 complex and 1024 points in t_1 and t_2, respectively, and 32 transients per t_1 point, for purified ^{15}N-labelled DEP monomer (at 298 K for **Fig. S2**, or at 283K for **Fig. S3**), or dimer (at 278 K; **Fig. S3**). In each case, 100 µM protein in aqueous phosphate buffer at physiological ionic strength (pH 6.7) was used. BEST-TROSY spectra were also obtained for ^{15}N-labelled DEP monomer or dimer after incubation with 300 µM of a 12-mer peptide from FZD5 (GKTLESWRRFTS) (Tauriello et al., 2012) which spans H8, but no chemical shift perturbation or line broadening was observed. Likewise, there was no observable chemical shift perturbation nor line broadening if the same 12-mer was ^{15}N-labelled as a Lip-tagged peptide (separated from the N-terminal tag by the linker ENLYFQS encoding a TEV cleavage site) and incubated at 100 µM with 300 µM purified DEP monomer. This indicates a low affinity between this H8 peptide and the DEP domain (K_d >1 mM), likely because the peptide contributes only a small number of residues to the whole interface between FZD5 and DEP (see main **Fig. 5C, D**), and maybe also because it lacks helical conformation which, in the equivalent peptide from FZD1, depends on association with micelles (Gayen et al., 2013).

SUPPLEMENTAL REFERENCES

Emsley, P., Lohkamp, B., Scott, W.G., and Cowtan, K. (2010). Features and development of Coot. Acta Crystallogr D Biol Crystallogr *66*, 486-501.

Favier, A., and Brutscher, B. (2011). Recovering lost magnetization: polarization enhancement in biomolecular NMR. J Biomol NMR *49*, 9-15.

Gayen, S., Li, Q., Kim, Y.M., and Kang, C. (2013). Structure of the C-terminal region of the Frizzled receptor 1 in detergent micelles. Molecules *18*, 8579-8590.

Langer, G.G., Hazledine, S., Wiegels, T., Carolan, C., and Lamzin, V.S. (2013). Visual automated macromolecular model building. Acta Crystallogr D Biol Crystallogr *69*, 635-641.

McCoy, A.J., Grosse-Kunstleve, R.W., Adams, P.D., Winn, M.D., Storoni, L.C., and Read, R.J. (2007). Phaser crystallographic software. J Appl Crystallogr *40*, 658-674.

Metcalfe, C., Mendoza-Topaz, C., Mieszczanek, J., and Bienz, M. (2010). Stability elements in the LRP6 cytoplasmic tail confer efficient signalling upon DIX-dependent polymerization. J Cell Sci *123*, 1588-1599.

Murshudov, G.N., Skubak, P., Lebedev, A.A., Pannu, N.S., Steiner, R.A., Nicholls, R.A., Winn, M.D., Long, F., and Vagin, A.A. (2011). REFMAC5 for the refinement of macromolecular crystal structures. Acta Crystallogr D Biol Crystallogr *67*, 355-367.

Sheldrick, G.M. (2010). Experimental phasing with SHELXC/D/E: combining chain tracing with density modification. Acta Crystallogr D Biol Crystallogr *66*, 479-485.

Stock, D., Perisic, O., and Lowe, J. (2005). Robotic nanolitre protein crystallisation at the MRC Laboratory of Molecular Biology. Prog Biophys Mol Biol *88*, 311-327.

Weierstall, U., James, D., Wang, C., White, T.A., Wang, D., Liu, W., Spence, J.C., Bruce Doak, R., Nelson, G., Fromme, P.*, et al.* (2014). Lipidic cubic phase injector facilitates membrane protein serial femtosecond crystallography. Nat Commun *5*, 3309.

Winn, M.D., Ballard, C.C., Cowtan, K.D., Dodson, E.J., Emsley, P., Evans, P.R., Keegan, R.M., Krissinel, E.B., Leslie, A.G., McCoy, A.*, et al.* (2011). Overview of the CCP4 suite and current developments. Acta Crystallogr D Biol Crystallogr *67*, 235-242.

Zeng, X., Tamai, K., Doble, B., Li, S., Huang, H., Habas, R., Okamura, H., Woodgett, J., and He, X. (2005). A dual-kinase mechanism for Wnt co-receptor phosphorylation and activation. Nature *438*, 873-877.
