# Supplementary material for: Wnt Signalosome Assembly by DEP Domain Swapping of Dishevelled
Source: Mol Cell. 2016 Oct 6;64(1):92–104. doi: 10.1016/j.molcel.2016.08.026 (PMC5065529; doi:10.1016/j.molcel.2016.08.026)
Supplement: Document S2. Article plus Supplemental Information [file mmc2.pdf]

Article

Molecular Cell

Wnt Signalosome Assembly by DEP Domain Swapping of Dishevelled

Graphical Abstract

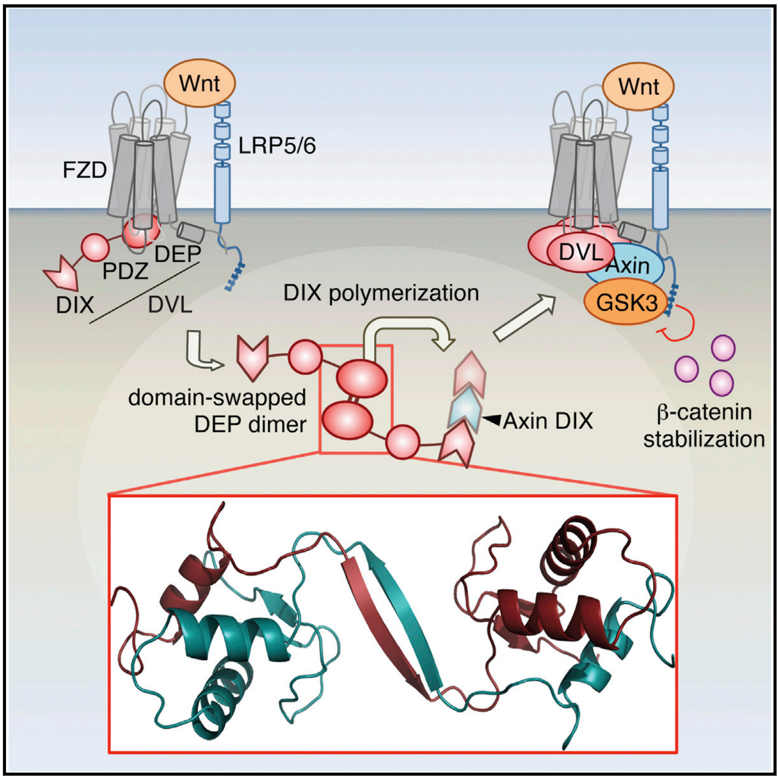

Highlights

- Wnt signalosome assembly by Dishevelled depends on DEP-dependent dimerization
- DEP dimerization via domain swapping favors unidirectional signaling
- DEP-dependent cross-linking of Dishevelled polymers triggers phase transition
- DEP dimerization is mutually exclusive with DEP-dependent binding to Frizzled

Authors

Melissa V. Gammons, Miha Renko, Christopher M. Johnson, Trevor J. Rutherford, Mariann Bienz

Correspondence

melissag@mrc-lmb.cam.ac.uk (M.V.G.),
mb2@mrc-lmb.cam.ac.uk (M.B.)

In Brief

Gammons et al. discover that Wnt signalosome formation by Dishevelled depends on dimerization by its DEP domain via domain swapping. This cross-links DIX-dependent Dishevelled polymers and, thus, promotes phase transition. They propose that DEP domain swapping triggered by high local concentration of Dishevelled in clathrin-coated pits initiates Wnt signal transduction.

Accession Numbers

5LNP
5SUZ
5SUY

Gammons et al., 2016, Molecular Cell *64*, 92–104
October 6, 2016 © 2016 MRC Laboratory of Molecular Biology.
Published by Elsevier Inc.
http://dx.doi.org/10.1016/j.molcel.2016.08.026

CrossMark

CellPress

Wnt Signalosome Assembly by DEP Domain Swapping of Dishevelled

Melissa V. Gammons,[1,2,*] Miha Renko,[1,2] Christopher M. Johnson,[1] Trevor J. Rutherford,[1] and Mariann Bienz[1,3,*]
[1]MRC Laboratory of Molecular Biology, Cambridge Biomedical Campus, Francis Crick Avenue, Cambridge CB2 0QH, UK
[2]Co-first author
[3]Lead Contact
*Correspondence: melissag@mrc-lmb.cam.ac.uk (M.V.G.), mb2@mrc-lmb.cam.ac.uk (M.B.)
http://dx.doi.org/10.1016/j.molcel.2016.08.026

SUMMARY

Extracellular signals are often transduced by dynamic signaling complexes ("signalosomes") assembled by oligomerizing hub proteins following their recruitment to signal-activated transmembrane receptors. A paradigm is the Wnt signalosome, which is assembled by Dishevelled via reversible head-to-tail polymerization by its DIX domain. Its activity causes stabilization of β-catenin, a Wnt effector with pivotal roles in animal development and cancer. How Wnt triggers signalosome assembly is unknown. Here, we use structural analysis, as well as biophysical and cell-based assays, to show that the DEP domain of Dishevelled undergoes a conformational switch, from monomeric to swapped dimer, to trigger DIX-dependent polymerization and signaling to β-catenin. This occurs in two steps: binding of monomeric DEP to Frizzled followed by DEP domain swapping triggered by its high local concentration upon Wnt-induced recruitment into clathrin-coated pits. DEP domain swapping confers directional bias on signaling, and the dimerization provides cross-linking between Dishevelled polymers, illustrating a key principle underlying signalosome formation.

INTRODUCTION

Cellular responses to external signals are often mediated by dynamic protein complexes that are assembled by cytoplasmic hub proteins following their recruitment to signal-activated transmembrane (TM) receptors (Li et al., 2012; Wu and Fuxreiter, 2016). Typically, these interactions are weak and reversible, and can be "head to tail," which results in open-ended polymerization, enabling hub proteins to assemble relatively large protein clusters without finite structures that are detectable as discrete puncta by light microscopy ("signalosomes") (Bienz, 2014). A salient molecular property of these signalosomes is their dynamicity, which renders them highly responsive to acute changes in cellular conditions: their hubs change phase between punctate and diffuse cytoplasmic within seconds, as judged by fluorescence recovery after photobleaching (FRAP) (e.g., Li et al., 2012; Schwarz-Romond et al., 2005). Thus, the polymerization of hub proteins increases their local concentration rapidly and dramatically, boosting their avidity for low-affinity binding partners, likely by lowering off-rates. This empowers them to interact with signaling effectors, even if these are present at a low cellular concentration.

One well-studied example is the Wnt signalosome, a dynamic signaling complex assembled by the hub protein Dishevelled upon binding of extracellular Wnt signals to its two TM receptors, low-density lipoprotein receptor-related protein 6 (LRP6) (Bilic et al., 2007) and Frizzled, a member of the large family of G-protein-coupled receptors (GPCRs) that transduce most signals in living systems (Venkatakrishnan et al., 2013). Wnt signalosome assembly relies on reversible head-to-tail polymerization by the N-terminal DIX domain of Dishevelled (Schwarz-Romond et al., 2007), which enables this hub to transduce Wnt signals to cytoplasmic effectors. These include β-catenin, which elicits transcriptional responses in dividing cells, but also "non-canonical" effectors (typically in non-dividing cells) that control fundamental properties of whole tissues, such as planar cell polarity (PCP) and convergent cell extensions (MacDonald et al., 2009). The Wnt > Dishevelled pathway is conserved from the most primate animals to humans; it specifies cell fates during normal embryonic development and in stem cell niches and can also cause disease if dysregulated; most notably, cancer (Clevers and Nusse, 2012).

Dishevelled was discovered in flies that have a single paralog (Dsh), whereas humans have three (DVL1–3). In addition to DIX, Dishevelled has a PDZ domain (Post-synaptic density protein-95, Disc large tumor suppressor, Zonula occludens-1) and a DEP domain (Dishevelled, Egl-10, and Pleckstrin), separated by long flexible linkers peppered with phosphorylation sites, and also a binding motif for the AP2 clathrin adaptor (Yu et al., 2010). Notably, AP2 and clathrin itself are crucial for signalosome assembly (Kim et al., 2013) and for non-canonical Wnt signaling (Yu et al., 2007), ascribing a key role to clathrin-coated pits in Wnt signal transduction (see Discussion).

The DIX domain is limited to the Wnt pathway, where it is also found in Axin, whose co-polymerization with Dishevelled via heterotypic DIX-DIX interactions triggers Wnt signal transduction (Fiedler et al., 2011). Its closest structural relative is the PB1 domain, found in numerous signaling hubs from yeast to mammals, which can also undergo dynamic head-to-tail polymerization (Bienz, 2014). Notably, polymerization by DIX or PB1 results in the assembly of one-dimensional

 © 2016 MRC Laboratory of Molecular Biology. Published by Elsevier Inc.
This is an open access article under the CC BY license (http://creativecommons.org/licenses/by/4.0/).

filaments (detectable by electron microscopy and biophysical methods); however, they manifest in cells as three-dimensional punctate structures (Schwarz-Romond et al., 2005). Thus, their formation requires additional molecular interactions that cross-link individual filaments (Sear, 2008), but these remain elusive.

Indeed, how signalosome polymerization is triggered by Wnt binding to its TM receptors remains a major unsolved question. An important step is, undoubtedly, the binding of Dishevelled to the intracellular face of Frizzled (FZD1–10 in humans; Fz1–4 in flies), which is pivotal for β-catenin-dependent and non-canonical Wnt responses (Bhanot et al., 1999). A highly conserved KTxxxW motif in the cytoplasmic tail of Frizzled is crucial for plasma membrane (PM) recruitment of Dishevelled and for signal transduction to β-catenin (Umbhauer et al., 2000) and to non-canonical Wnt effectors (Wu et al., 2008). Structurally, this motif maps to a short amphipathic α-helix (H8) found in most known GPCR structures, including Smoothened (Wang et al., 2013), a Frizzled relative whose function is dedicated to Hedgehog signal transduction (Schulte and Bryja, 2007). Biophysical in vitro assays revealed interactions between this motif and Dishevelled PDZ (Wong et al., 2003) and DEP (Tauriello et al., 2012), but whether these are needed for signalosome assembly in cells is unknown. Notably, Frizzled-dependent PM translocation of Dishevelled is blocked by a lysine-to-methionine mutation in DEP (e.g., Axelrod et al., 1998; Yu et al., 2007), mimicking *Drosophila dsh^1*, which causes PCP defects in fly tissues (Axelrod et al., 1998; Boutros et al., 1998), implicating the DEP-Frizzled interaction in Wnt signal transduction by Dishevelled. In the absence of Wnt, the DEP domain facilitates the ubiquitylation of Frizzled by the E3 ubiquitin ligase ZNRF3, which promotes the clathrin-dependent endocytosis of Frizzled and subsequent lysosomal degradation (Jiang et al., 2015), presumably safeguarding against fortuitous Frizzled-mediated signaling prior to Wnt signaling. Despite these fundamental roles of Dishevelled DEP prior to and following Wnt signaling, there is no clear understanding of its intrinsic molecular function or, indeed, of any DEP domain in other proteins whose roles in signal transduction seem curiously different in each case (Consonni et al., 2014).

To pin down this function, we undertook a detailed structure-function analysis of DVL2 DEP. Thus, we discovered that DEP dimerization is crucial for the assembly of functional signalosomes. Interestingly, DEP dimerization is based on domain swapping, as revealed by its crystal structure, whereby a DEP monomer extends its N-terminal α-helix to donate this to a second DEP recipient. Cell-based functional tests of dimerization mutants indicate that monomeric DEP binds to Frizzled, and we propose that DEP dimerization is triggered by a local high concentration following Wnt-dependent relocation of Dishevelled into clathrin-coated pits. The primary function of the DEP-dependent dimerization is the provision of cross-links between DIX polymers—an a priori requirement for the assembly of linear filaments into dynamic "punctate" phase-separated protein assemblies (Li et al., 2012), sometimes also called liquid droplets (Wu and Fuxreiter, 2016; Brangwynne et al., 2009).

RESULTS

DEP Dimerization Is Necessary and Sufficient for Signalosome Formation

DIX-DIX interactions are weak (K_d mid-micromolar) (Bienz, 2014); they cannot occur at the sub-micromolar physiological concentrations of Dishevelled typically found in cells. Indeed, co-immunoprecipitation (coIP) between differently tagged DVL2 proteins is efficient, even if these lack the DIX domain (ΔDIX), and we mapped this additional (strong) interaction to the DEP domain whose deletion (ΔDEP) abolished the coIP between DVL2-GFP and FLAG-DVL2 (Figure 1A). Similar coIP assays with DVL2 truncations, and between ΔDEP and minimal DVL2 or *Drosophila* Dsh DEP, confirmed that DEP is necessary and sufficient for Dishevelled dimerization (Figure S1, available online).

Remarkably, the DEP domain is also required for assembly of Wnt-independent Dishevelled signalosomes in cells (Figure 1B): distinct cytoplasmic puncta are observed in transfected HeLa cells overexpressing wild-type (WT) DVL2-GFP, but neither in a polymerization-deficient DIX mutant (M2M4) (Schwarz-Romond et al., 2007), nor in cells expressing ΔDEP-GFP, although in the latter case, some puncta can be seen in a low fraction of transfected cells (Figure 1B). Since DVL2 needs to be punctate to recruit Axin (Bienz, 2014), we expected ΔDEP-GFP to be dysfunctional in signaling. This is the case: the signaling activity of ΔDEP-GFP, as measured by a β-catenin-dependent transcriptional reporter (called SuperTOP), is significantly reduced (Figure 1C), confirming previous results (e.g., Rothbächer et al., 2000). Its residual activity reflects DIX-dependent polymerization, which can evidently bypass its DEP dependence under overexpression conditions, in contrast to the polymerization-blocking mutant, which eliminates puncta (Figure 1B) and signaling to β-catenin (Schwarz-Romond et al., 2007) (Figure 1C). Hereinafter, we shall use the term "signaling" to refer to β-catenin-dependent transcriptional activity.

We previously found that the substitution of DIX with a heterologous dimerization domain (called TPR, a leucine zipper from an oncogenic met receptor) failed to restore puncta formation and signaling of DVL2ΔDIX (Schwarz-Romond et al., 2007). However, substituting DVL2 DEP with TPR (ΔDEP > TPR) restored self-interaction in coIP assays (Figure 1D), as well as efficient puncta formation (Figure 1E) and signaling, above the level of WT DVL2 (Figure 1F). Notably, this DEP function is conserved in *Drosophila*, since the Dsh DEP domain fully substitutes for the activity of its human counterpart (Figures 1D–1F), while the DEP domains from the unrelated signaling proteins EPAC and Pleckstrin do not (M.V.G., unpublished data). Thus, two key mechanistic principles empower DVL2 to form functional signalosomes: polymerization by DIX, and dimerization by DEP, whereby the latter is required for the former to occur in cells.

DEP Forms a Domain-Swapped Dimer

Previous solution and crystal structures of DEP (Wong et al., 2000; Yu et al., 2010) revealed a small globular fold whose hydrophobic core is formed by three α helices (H1–H3; Figure 2A). These structures are almost identical, except for the orientation of a prominent loop (containing two β strands, β1 and β2, to be

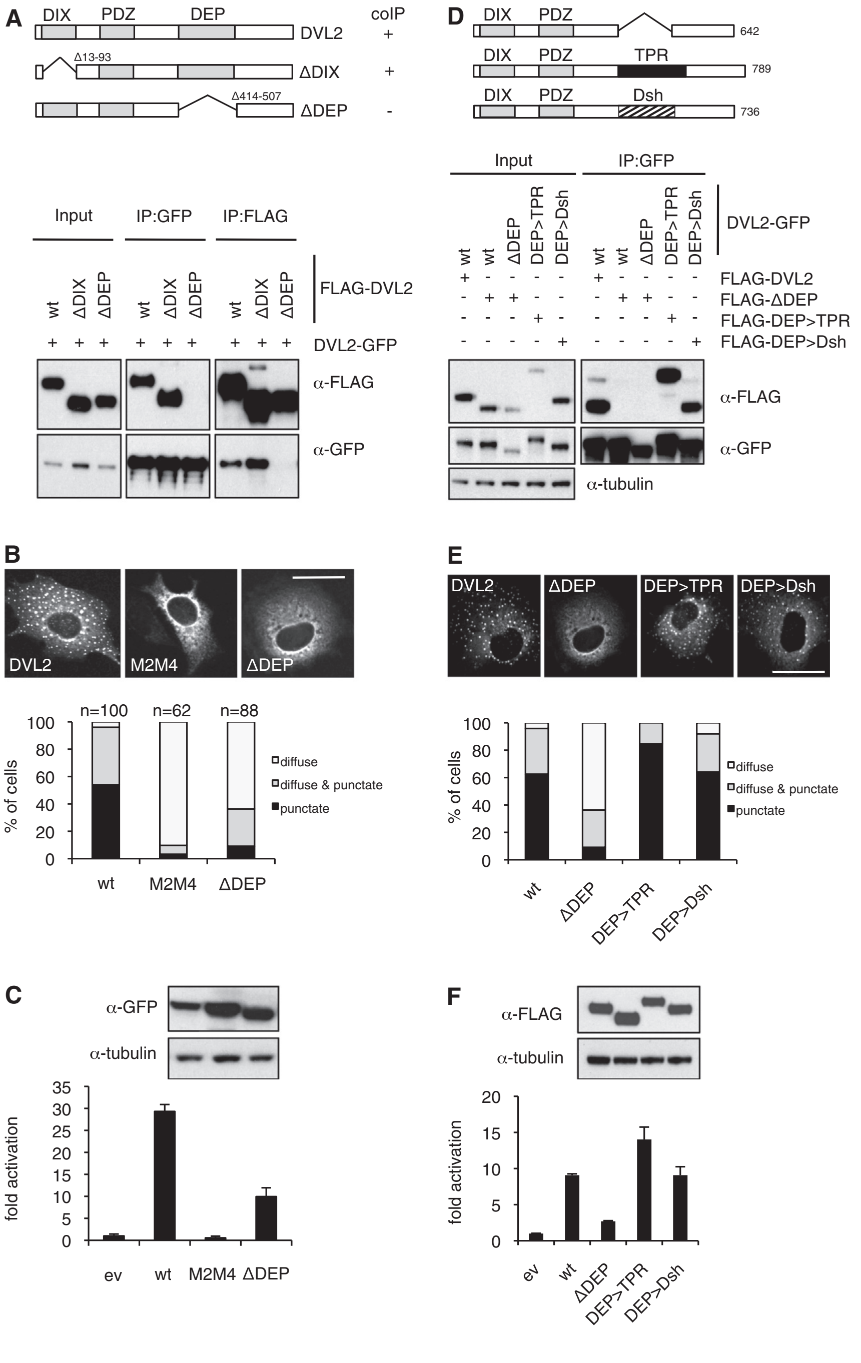

Figure 1. Assembly of Functional Signalosomes by DEP Dimerization

(A) Western blots of immunoprecipitates (IPs) of FLAG-DVL2 or DVL2-GFP (resin indicated above panels) after co-expression in HEK293T cells (lysed 24 hr after transfection), probed with antibodies as indicated on the right; above, cartoon of DVL2 constructs and summary of binding. wt, wild-type.

(B) Confocal images of representative HeLa cells (fixed 18 hr after transfection), co-expressing WT or mutant DVL2-GFP as indicated; below, quantitative analysis of transfected cells (classed as predominantly diffuse, diffuse plus punctate, or predominantly punctate). n, number of cells scored.

(C) SuperTOP assays of HEK293T cells, expressing WT or mutant DVL2-GFP (as indicated; above, corresponding western blot). ev, empty vector control.

(D) CoIP assays as in (A), confirming DVL2 self-association via TPR or Dsh DEP (hatched).

(E) Confocal images and quantitation of results as in (B) (100 cells scored).

(F) SuperTOP assays as in (C).

Error bars indicate SEM of more than three independent experiments. Scale bars, 10 μm. See also Figure S1.

called "DEP finger") that projects outward from the core; a second shorter loop (containing β3 and β4) at the C terminus of H3 is tucked under the DEP core (Figure 2B, magenta). We used these structures to design 31 point mutations (mostly in surface-exposed residues) to block dimerization, but none of these reduced DEP coIP (Table S1), suggesting an extensive DEP-DEP interface. This was somewhat surprising, given the compact fold of DEP.

Therefore, we took a structural approach to determine the DEP-DEP interface. Purifying lipoyl-tagged DEP (Lip-DEP$_{416–511}$) by size exclusion chromatography (SEC), we observed two main peaks, corresponding to monomers and dimers (discussed later), in addition to a higher mass species. Thus, we crystallized the monomeric and dimeric fractions and determined three structures at 1.84–2.06 Å resolution (Table S2). Remarkably, each structure exhibits a swapped dimer, regardless of the crystal form: namely, molecule A (Figure 2B, turquoise) donates H1 to the H2–H3 core of molecule B (Figure 2B, light turquoise) by extending loop 1, and vice versa. Thus, two DEP cores are connected by an extended β sheet, composed of two antiparallel β strands (each made up by a β1β2 tandem) that interact with each other through backbone atoms of residues 442–447 (Figures 2A and 2B). This dimer-specific interface is based on six new hydrogen bonds, which may bias the equilibrium toward the domain-swapped configuration. Indeed, dimerization does not involve any loss of intramolecular interactions, since β1 and β2 do not interact detectably in the monomer (Wong et al., 2000). The relative configurations of the α helices are basically the same in the monomer and in our domain-swapped dimer structures, with a root-mean-square deviation (RMSD) of <2.04 Å between monomer and dimer backbones (84 aligned Cα atoms).

Interestingly, the solvent-exposed "underside" of the connecting β sheet presents a hydrophobic patch, made up of a triad of hydrophobic amino acids (M443, L445, and I447) located at

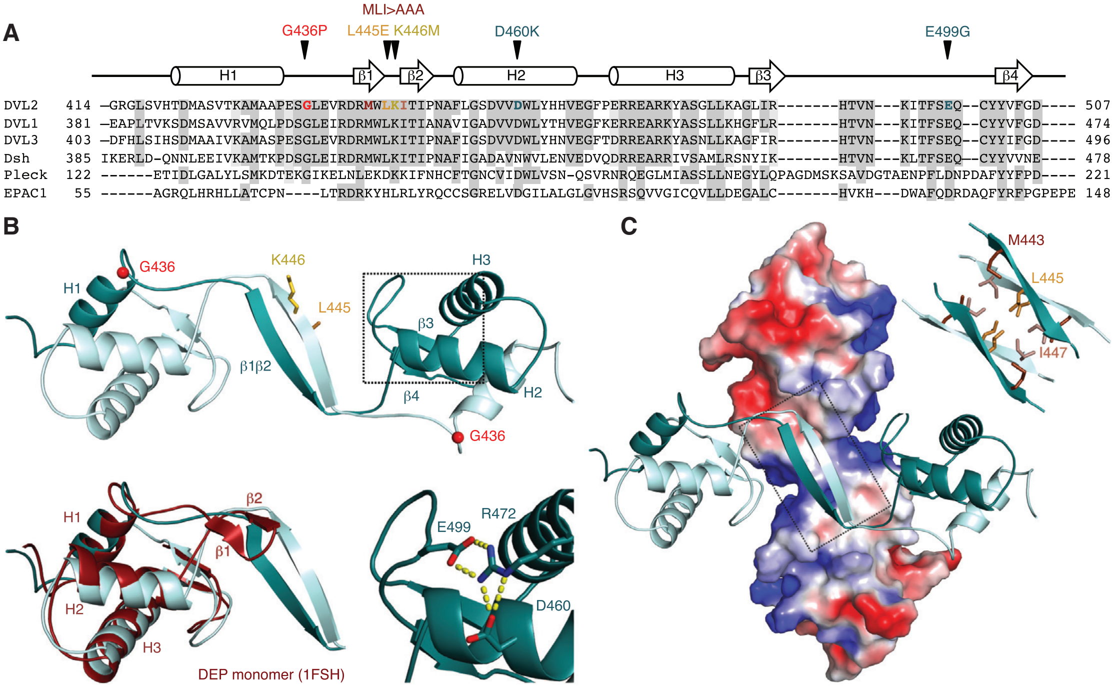

Figure 2. DEP Dimerization by Domain Swapping

(A) Sequence alignment of DEP domains (all human except for Dsh DEP); indicated are secondary structure elements (above) and conserved (highlighted) and mutated (colored) residues.

(B) Top: structure of DEP dimer (in ribbon representation), revealing domain swapping. Secondary structure elements are labeled in molecule A (dark turquoise); L445 and K446 are shown (in stick) in molecule B (light turquoise). Bottom: structure is superimposed on DEP monomer (maroon). Inset, salt-bridge network, with key residues in stick (red indicates oxygen; blue indicates nitrogen).

(C) Structure of DEP tetramer (top dimer as in B; bottom dimer in electrostatic surface representation); inset, hydrophobic triad (residues in stick, labeled in molecule A).

See also Tables S1 and S2.

the DEP finger tip of the monomer (Figures 2A and 2C). In each crystal, two DEP dimers associate with each other through this hydrophobic patch, thus linking four DEP cores into a cross-shaped tetramer (Figure 2C). Presumably, the high protein concentration in the crystallization drops favored the tetramerization of DEP. According to PISA (protein interfaces, surfaces, and assemblies) (Krissinel and Henrick, 2007), the DEP tetramer should be stable in solution.

To test this, we used SEC-MALS (SEC with multi-angle light scattering). Thus, we confirmed that the molecular masses of the unfractionated and purified fractions correspond to monomers, dimers, and tetramers (Figure 3A; molar ratios, 4:2:1). At 4°C, the dimers proved to be stable, without any detectable dissociation over the course of several days. The hydrodynamic radius of the dimer determined during SEC-MALS confirmed that its shape in solution is larger than a typical globular fold of 44 kDa mass (Figure 3B), consistent with the extended configuration of the swapped dimer seen in the crystal.

The DEP-DEP interface is vast (burying 5,020 $Å^2$) and elongated, as further supported by nuclear magnetic resonance (NMR) experiments: we observed pronounced exchange broadenings of numerous cross-peaks in BEST-TROSY ^1H-^{15}N correlation spectra of ^{15}N-labeled DEP dimers in solution, whereas the spectrum of ^{15}N-labeled monomer shows well-defined cross-peaks of even intensity, as reported previously (Wong et al., 2000). Overlay of the monomer and dimer spectra (following peak assignments; Figure S2) revealed that the peaks with strongest exchange broadening are located predominantly in the β sheet between the two DEP cores (Figure S3).

Dimerization-Defective DEP Mutants Fail to Signal

To determine whether the DEP-dependent dimerization of DVL2 is required for its signaling activity, we adopted a strategy that was used extensively in other proteins to block domain swapping (Rousseau et al., 2003), mutating the glycine at the base of the DEP loop to proline (G436P), to attenuate the extension of this loop needed for H1 exchange (Figure 2B). Indeed, G436P produces a folded soluble protein whose conformation is shifted significantly toward the monomeric state (Figures 3C and 3D). When introduced into full-length DVL2, G436P attenuates coIP in transfected HEK293T cells (Figure 4A) and reduces signaling and puncta formation to background levels, similarly to ΔDEP (Figures 4B and 4C). In the rare escaper cells that exhibit G436P puncta, these invariably fail to co-localize with FLAG-Axin (Figure 4D), and coIP with FLAG-Axin is barely detectable (Figure S4), explaining why G436P fails to signal.

Notably, G436P is also unphosphorylated (Figure 4B), like two other DEP point mutations, E499G and D460K (Mund et al.,

A

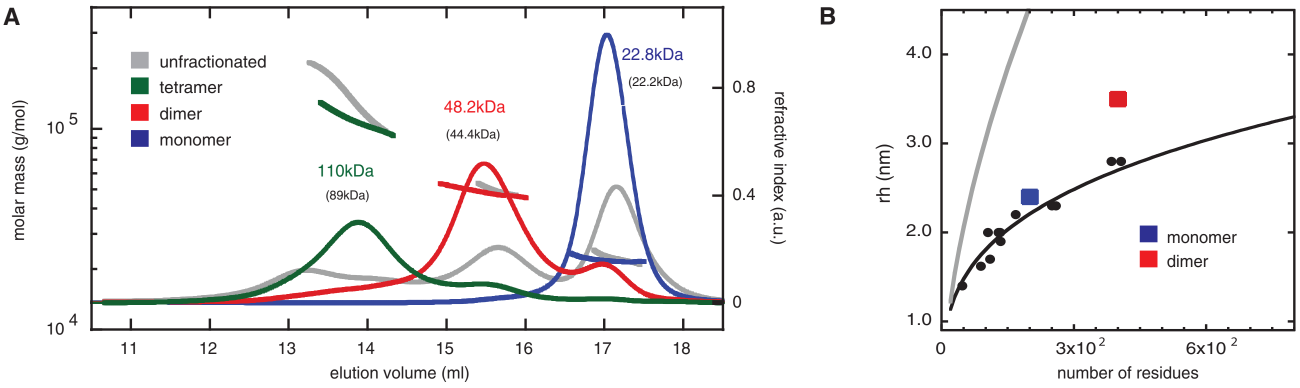

C

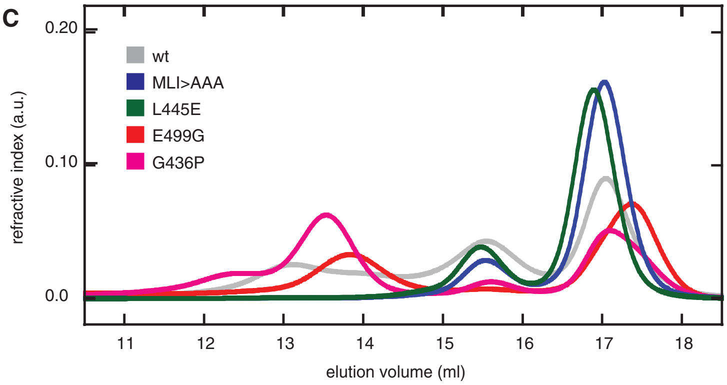

D

mutant	monomer	dimer	tetramer
wt	++	++	+
K446M	++	++	+
D460K	++	-	+
G436P	++	-	+
E499G	++	-	+
L445E	++	++	-
MLI>AAA	++	+	-

Figure 3. SEC-MALS of Purified DEP Domain

(A) Elution profiles of unfractionated (gray) Lip-DEP$_{416–511}$ revealing multiple species, or of monomeric (blue), dimeric (red), or tetrameric (green) Lip-DEP$_{416–511}$ after purification, and calculated average molecular masses of peak material (above peaks; numbers in brackets indicate expected molecular masses).

(B) Analysis of protein hydrodynamic radius (rh), indicating a globular fold of DEP monomer (blue), but an extended conformation of DEP dimer (red); black and gray lines indicate rh values based on protein size proposed for folded globular and unfolded proteins (Wilkins et al., 1999).

(C) Elution profiles of wild-type (wt) and mutant Lip-DEP$_{416–511}$.

(D) Summary of results.

See also Figures S2 and S3.

2015). Dishevelled is heavily phosphorylated upon Frizzled association, and also when overexpressed, which has been ascribed to DEP function (Rothbächer et al., 2000), and although the physiological relevance of this modification remains unproven (Bernatík et al., 2014), it provides a useful hallmark of signaling competence. Indeed, neither E499G nor D460K signal (Figure 4B), and both mutations cause predominantly diffuse GFP fluorescence, in some cases superimposed by puncta (Mund et al., 2015) (Figure 4C), but these rarely co-localize with co-expressed FLAG-Axin (Figure 4D). Furthermore, FRAP assays revealed that they recover more slowly, and less fully, than WT DVL2-GFP puncta, and the two mutations also fail to co-immunoprecipitate with FLAG-Axin (Figure S4). Thus, the E499G and D460K puncta are abnormal protein assemblies that neither interact with Axin nor signal. Importantly, like G436P, these two mutations severely attenuate dimerization of purified DEP in solution, shifting its equilibrium toward monomeric (Figures 3C and 3D). The disability of these mutants to dimerize explains their defects in signaling.

The dimerization defect resulting from mutating E499 and D460 can be rationalized by a striking salt-bridge network between their negatively charged side chains and the guanidinium group of a key arginine (R472; Figure 2B, inset). These salt bridges constrain the rotamer conformation of R472, which is tilted toward E499G and D460K (rather than projecting freely into the solvent). Notably, E499 is located on the loop between β3 and β4, and its salt bridge with R472 appears to be mainly responsible for tucking this loop under the DEP core. Removing this key interaction might predispose this loop to “flapping,” which could attenuate dimerization and destabilize the DEP core. This key interaction between E499 and R472 is likely to be affected by D460K, albeit indirectly, via a destabilizing effect of this mutation on the whole salt-bridge network, which may explain why D460K is slightly less dysfunctional than E499G in some of our assays. We cannot determine whether this salt-bridge network also forms in the DEP monomer because the resolution of the monomer structures is too low, but it appears dispensable for the stability of the monomeric fold, judging by

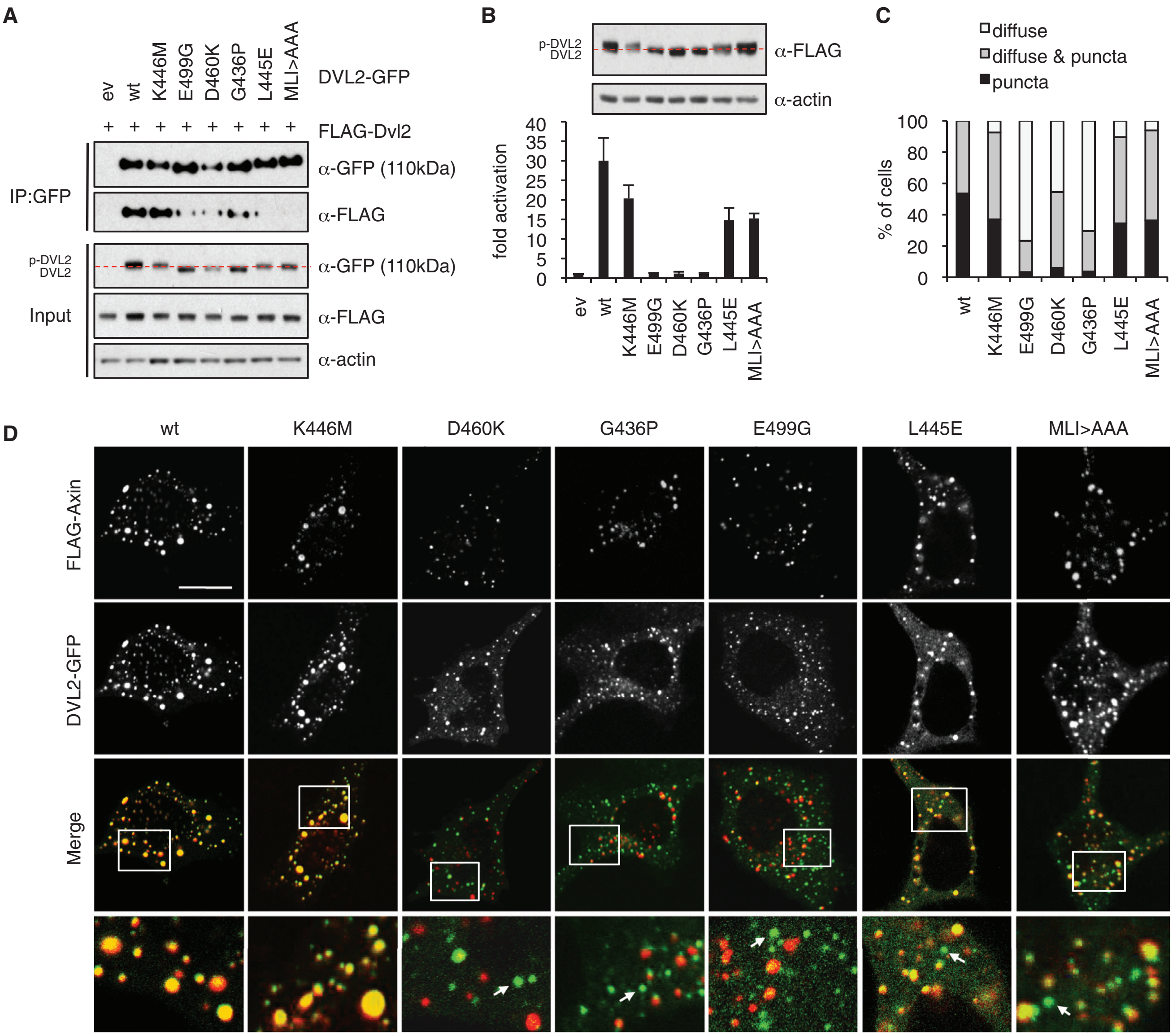

Figure 4. DEP Dimerization Mutants Fail to Assemble Functional Signalosomes

(A) CoIPs as in Figure 1A, confirming reduced self-association of dimerization (D460K, E499G, and G436P) and tetramerization (L445E, MLI > AAA) mutants; p-DVL2, phosphorylated DVL2 (above red dotted lines).

(B) SuperTOP assays and corresponding western blot, as in Figure 1C.

(C) Quantitation of transfected HEK293T cells, as in Figure 2B (for results from HeLa cells, see Figure S4).

(D) Confocal images of representative HeLa cells co-expressing DVL2-GFP (green) and FLAG-Axin (red), fixed and stained with α-FLAG antibody 18 hr after transfection; arrows in merged mark DVL2 puncta without Axin.

Error bars indicate SEM of more than three independent experiments. Scale bars, 10 μm. See also Figure S4.

the relatively conservative changes in the NMR spectra of E499G and D460K compared to WT (Figure S2).

To test whether DEP tetramerization is required for DVL2 signaling, we designed a triple-alanine mutation of the hydrophobic triad (MLI > AAA), and a substitution of the central triad residue to glutamic acid (L445E), which should repel tetramerization. We confirmed by SEC-MALS that both mutations block tetramerization of purified DEP, although neither affects dimerization (Figures 3C and 3D). In coIP assays, the level of DVL2 self-association is reduced to background levels (Figure 4A), indicating that the DEP dimers formed by these mutants in vitro (Figures 3C and 3D) are not stable in vivo. Their signaling activities are somewhat reduced (Figure 4B); nevertheless, they form puncta, albeit superimposed on a slightly higher level of diffuse fluorescence compared to DVL2-GFP (Figures 4C and 4D), signifying dysfunctional signalosome assembly. Also,

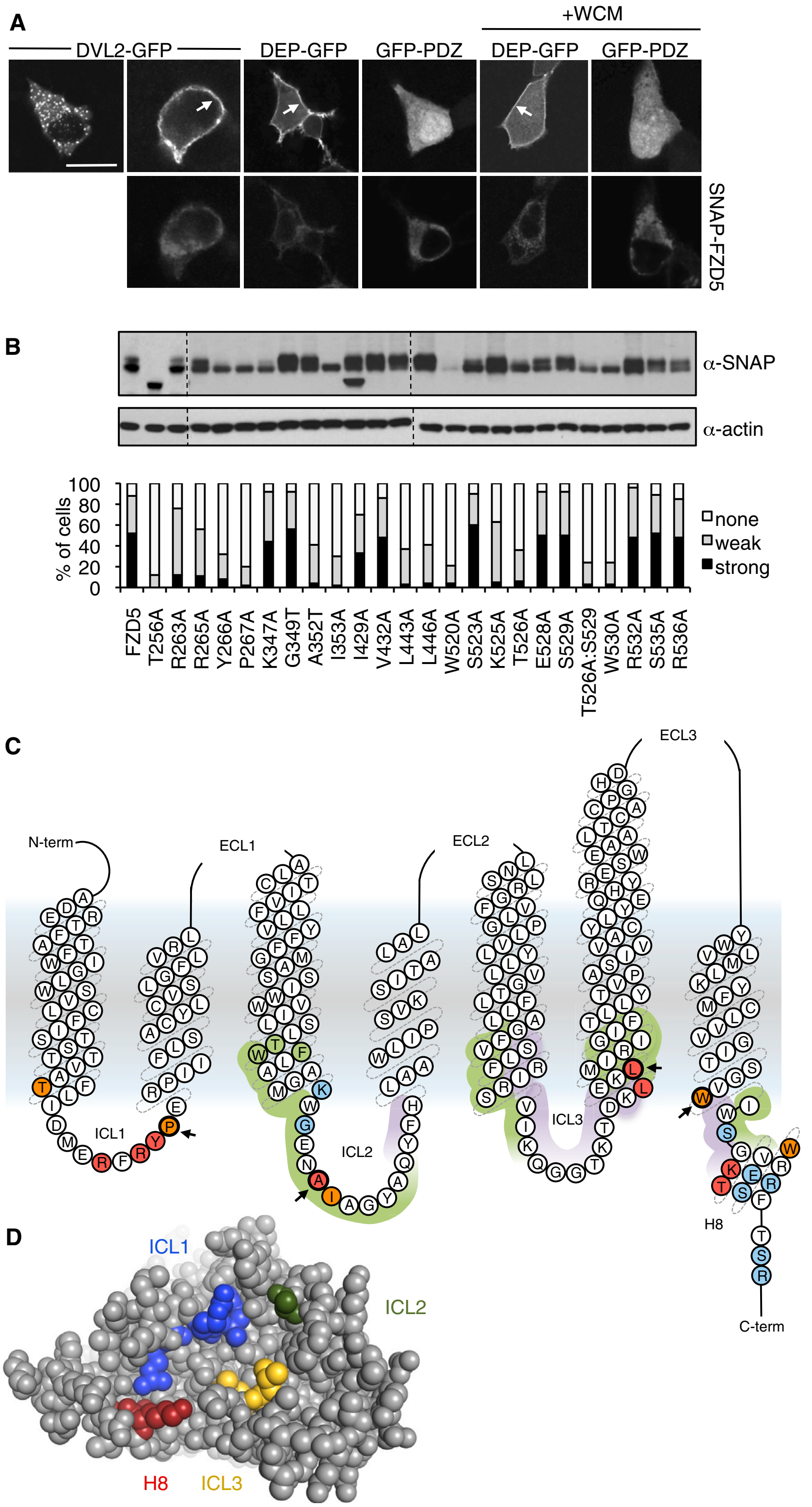

Figure 5. FZD5 Residues Required for DEP Recruitment

(A) Confocal images of HeLa cells, co-expressing DVL2-GFP, GFP-PDZ, or DEP-GFP (top) with SNAP-FZD5 (bottom), fixed and stained with α-SNAP antibody as in Figure 4D; WCM, Wnt3a-conditioned medium (30 min before fixation); arrows indicate FZD5-dependent PM recruitment. Scale bar, 10 μm.

(B) Quantitation of FZD5-dependent PM recruitment of wild-type (wt) or mutant DEP-GFP (100 cells scored in each case) and corresponding western blots (from two independent experiments, juxtaposed as indicated by dotted lines; orange indicates low expression, lack of modification, or proteolysis).

(C) Cartoon of FZD5; highlighted are residues required (red or orange if abnormally expressed or unmodified) or dispensable (turquoise) for DEP recruitment (see B). Arrowheads indicate *fz* or *fz2* alleles; bracketed are regions contacting Arrestin (purple) or Gs (green) in class A GPCRs (see Figure S5). TWF (green), topological equivalent of DRY; term, terminal.

(D) On-view of putative DEP interface of FZD5 (modeled on PDB: 4JKV; see Figure S5), as defined by residues required for DEP recruitment (red in B, colored in rainbow according to location).

some proportion of the L445E and MLI > AAA puncta fail to recruit FLAG-Axin (Figure 4D, "Merge" panels), consistent with the attenuated signaling activity of these mutants. Nevertheless, DEP-dependent tetramerization is clearly less important than dimerization for signaling by Dishevelled, consistent with our result that its dimerization by a heterologous module restores efficient signaling of ΔDEP (Figures 1E and 1F).

DEP Recruitment Requires an Extensive Intracellular Surface of Frizzled

DVL1 DEP binds to the KTxxxW motif and to the adjacent intracellular loop 3 (ICL3) of FZD5 (Tauriello et al., 2012). We decided to further refine this binding site by adopting the same FZD-dependent membrane translocation assay in transfected HEK293T cells. We confirmed that the minimal DEP domain ($DEP_{414–507}$-GFP) is recruited to co-overexpressed PM-associated SNAP-FZD5 as efficiently as DVL2-GFP (Figure 5A) and that this recruitment is blocked by mutations in KTxxxW (Tauriello et al., 2012) (Figure 5B). Notably, T526A does not affect in vitro binding of purified Dvl PDZ to FZD7 KTxxxW peptides (Punchihewa et al., 2009), arguing against PDZ being the functionally relevant ligand of this motif in vivo. Indeed, we cannot detect any FZD5-mediated PM recruitment of a minimal DVL2 PDZ domain ($PDZ_{248–353}$-GFP), even after stimulation with Wnt3a (Figure 5A), as previously shown (Pan et al., 2004; Tauriello et al., 2012),

confirming DEP rather than PDZ as the DVL2 domain binding to Frizzled. This is fully consistent with previous studies that internal DEP, but not PDZ, deletions of Dishevelled abolish its PM recruitment and signaling to β-catenin (Axelrod et al., 1998; Rothbächer et al., 2000). Our results further confirm that the DEP-Frizzled interaction does not rely on Wnt binding to Frizzled (Jiang et al., 2015). Indeed, DEP recruitment to FZD5 is less pronounced even after a short pulse of Wnt3a (Figure 5A), indicating that Wnt binding to Frizzled weakens its interaction with DEP.

We generated an additional 20 alanine (or threonine) substitutions in FZD5, guided by a systematic alanine scanning screen that identified residues in rat Fzd1 required for signaling in *Drosophila* S2 cells (Cong et al., 2004), by *fz* and *fz2* alleles isolated in genetic screens in *Drosophila* (Povelones et al., 2005; Strutt et al., 2012), while also re-testing some previous FZD5 mutants (Tauriello et al., 2012). We thus confirmed that L443A and L446A (in ICL3; Figure 5C) block DEP recruitment to FZD5, although mutations of I429 and V432 in the upstream segment of this loop had no effect in our hands (Figure 5B). We also identified additional residues in ICL1 and ICL2 that block, or significantly reduce, DEP recruitment to FZD5, while other residues within or flanking H8 had no effect (Figures 5B and 5C). Also dispensable are residues near the base of TM3, which, in class A GPCRs, engages in direct contact with Gs (Carpenter et al., 2016; Rasmussen et al., 2011) (Figures 5C and S5), suggesting that G proteins are not required for DEP binding to Frizzled (see also Discussion).

Five of the FZD5 residues whose mutation disabled DEP recruitment also exhibited defects in expression or modification of FZD5 (Figure 5C, orange), most notably, two (T256A and W520A) whose topological equivalents in Smoothened engage in a key structural interaction (Wang et al., 2013): T256A fails to give rise to full-length product, and W520A is barely expressed (Figure 5B, top). Other mutants lack the slowly migrating species seen with WT FZD5 (Figure 5B, top) likely to reflect glycosylated FZD5 and, thus, may not traffic normally through the endoplasmic reticulum and Golgi compartment to the cell surface. This could explain their failure to recruit DEP, given that only the PM-associated FZD5, but not the cell-internal FZD5 pool, is able to recruit DVL2 or DEP (Figure 5A). However, the remaining eight mutations (Figure 5C, red) do not majorly affect the expression or modification of FZD5, and thus delineate its DEP binding site, which spans the base of TM7 and proximal H8, plus adjacent residues from all three intracellular loops (Figure 5D). Notably, seven of these residues are either large hydrophobic or invariant among human FZD paralogs, but four of them are distinct in Smoothened (Figure S5). This explains why Smoothened recruits neither DEP-GFP nor DVL2-GFP in our assays (M.V.G., unpublished data), consistent with the genetic evidence that Dishevelled does not transduce Hedgehog signals.

DEP Dimerization Is Dispensable for Dishevelled Binding to Frizzled

Next, we asked whether the Frizzled-DEP interaction depends on DEP dimerization. Our attempts to investigate this interaction by NMR were unsuccessful (see Supplemental Information). Therefore, we resorted to a previously developed blocking assay (Pan et al., 2004; Tauriello et al., 2012), which was used to demonstrate that overexpressed DEP, but neither PDZ nor DIX, blocks the Wnt-dependent signaling activity of endogenous Dishevelled. Indeed, overexpressed DEP-GFP reduces the signaling of endogenous DVL to ~25% of the GFP controls (Figure 6A), consistent with the robust interaction of DEP-GFP with FZD5 (Figure 5A).

Strikingly, the DEP dimerization mutants proved fully active in blocking signaling by endogenous DVL, while the tetramerization mutants failed to do so (Figure 6A). In support of this, the dimerization mutants are recruited to FZD5 as efficiently as WT DEP-GFP, while the tetramerization mutants remain cytoplasmic (Figure 6B). We noticed that the residues mutated in the latter map to a single coherent patch on the "palm-facing" surface of the DEP finger in the monomer, with L445 at its tip (Figure 6C). Its neighbor, K446M (mimicking *dsh*[1]), also fails to block signaling by endogenous DVL (Figure 6A), consistent with previous results (e.g., Tauriello et al., 2012), as do alanine substitutions of W444 and R442, solvent-exposed residues that are located further proximally on the DEP finger (Figures 6A and 6C). Together, these mutants define the DEP finger as a key structural element mediating Frizzled binding. Remarkably, the very same element is crucial for tetramerization (Figures 3C and 3D), indicating that DEP tetramerization and Frizzled binding are mutually exclusive (see Discussion). Indeed, DEP dimerization is clearly dispensable in these assays (Figures 6A and 6B), implying that the DEP domain binds to Frizzled as a monomer.

DISCUSSION

We discovered a conformational switch of Dishevelled based on DEP domain swapping, which is essential for the assembly of functional signalosomes and for Wnt signal transduction to the nucleus. DEP-dependent dimerization of Dishevelled leads to tetramerization, which is mutually exclusive with binding to Frizzled. It also boosts the local concentration of the linked DIX by at least 4-fold, which increases its avidity for Axin DIX, facilitating hetero-polymerization between the two DIX domains—the key step initiating stabilization of β-catenin. DEP-dependent domain swapping is also critical for Wnt-dependent signaling activity in complementation assays based on physiological re-expression of DVL2 in DVL triple-knockout cells (M.V.G., unpublished data; see Figure S6 for full reference details). Integrating our results with previous discoveries of the pivotal role of AP2μ and clathrin in Wnt signaling (Kim et al., 2013; Yu et al., 2007), we propose a mechanistic model according to which clathrin-coated pits initiate signalosome assembly and Wnt signal transduction by catalyzing domain swapping by Dishevelled DEP (Figure 7).

Clathrin-Coated Pits as Locales for Wnt Signalosome Assembly

The heterotetrameric AP2 clathrin adaptor associates with the PM by binding to phosphatidylinositol-4,5-phosphate ($PtdIns4,5P_2$) via its two large subunits (α and β) and recruits clathrin (via β) to form clathrin-coated pits (Kirchhausen et al., 2014; Owen et al., 2004). $PtdIns4,5P_2$ binding induces a conformational change of its μ subunit, allowing it to bind cargo. One such cargo is LRP6, which binds to AP2μ via a highly conserved YxxΦ motif in its cytoplasmic tail; like clathrin itself, AP2μ is

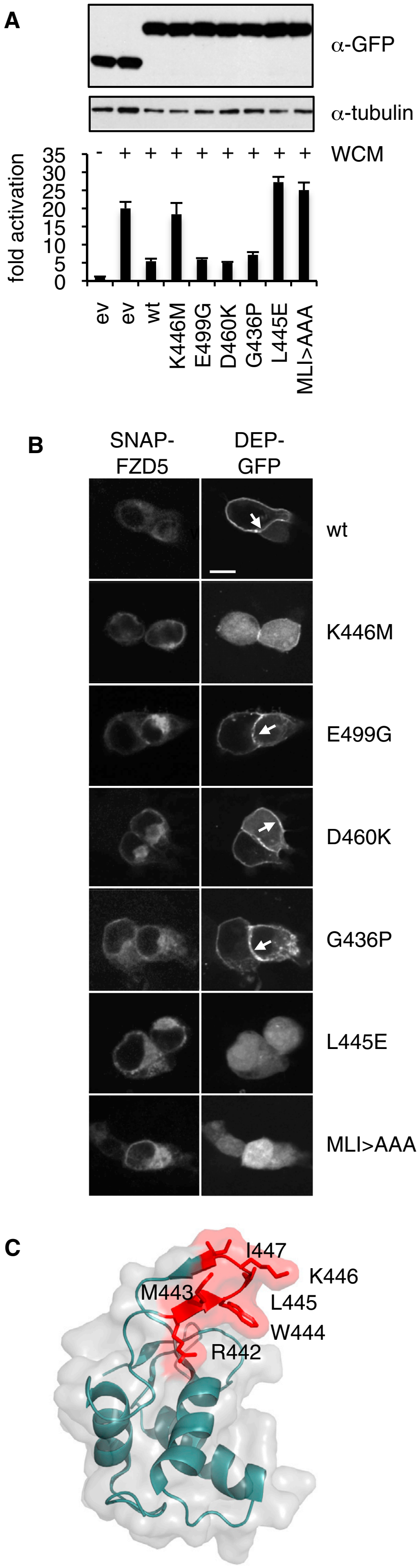

Figure 6. DEP Dimerization Is Dispensable for FZD5 Binding

(A) SuperTOP assays and corresponding western blot, as in Figure 1C (+ WCM indicates treatment as in Figure 5A). ev, empty vector control; wt, wild-type.

(B) Confocal images of HeLa cells, as in Figure 5A; arrows indicate FZD5-dependent PM recruitment.

(C) Structure of DEP monomer (Wong et al., 2000); red indicates loop 1 residues (in stick) required for blocking activity in (A).

Error bars indicate SEM of more than three independent experiments. Scale bar, 10 μm.

essential for signalosome assembly and Wnt signal transduction to β-catenin (Kim et al., 2013). Clathrin's binding to AP2β provides a second (indirect) molecular link between AP2 and LRP5/6, resembling a so-called chelate effect. Chelate effects consolidate the transient weak interactions between clathrin, its adaptors, and their cargoes (Owen et al., 2004) and, thus, promote the growth of clathrin-coated pits whose maturation is reinforced by further interaction networks between their components until they eventually pinch off as vesicles (Kirchhausen et al., 2014). The association between LRP5/6 and AP2-clathrin is biochemically stable (Kim et al., 2013), suggesting that LRP5/6 is poised as a linchpin in nascent pits to transduce Wnt signals to β-catenin. By contrast, Frizzled does not contain any recognizable motifs that would allow it to associate with AP2 or clathrin directly. However, Frizzled could be relocated to pit-associated LRP6 by Wnts that bind simultaneously to the extracellular domains of Frizzled and LRP6 (e.g., Chu et al., 2013). Indeed, linking Frizzled to LRP5/6 may be the single most important function of Wnts in triggering signal transduction (discussed later).

Dishevelled can also bind to μ2 through a conserved YxxΦ motif downstream of DEP (Yu et al., 2010) (Figures 2A and 2B), which facilitates non-canonical Wnt signaling (Yu et al., 2007). However, this interaction appears to be weak (Yu et al., 2010) and does not sustain a biochemically stable complex with LRP6-containing signalosomes (Bilic et al., 2007), and it may, therefore, only permit a fleeting association of Dishevelled with clathrin-coated pits. Furthermore, we cannot detect any functional requirement for the μ2-binding YHEL motif in any of our assays, including those dependent on Wnt (Figures S6 and S7), arguing against this interaction mediating the recruitment of Dishevelled to pit-associated LRP6. Rather, we envisage that Dishevelled is relocated passively to clathrin-coated pits, via binding to Frizzled. We note that, once recruited to these pits, Dishevelled can provide further chelation by increasing the local concentration of PtdIns4,5P_2 by interacting with PtdIns-4-phosphate-5-kinase-1 and activating it toward its substrate PtdIns4P (Pan et al., 2008; Hu et al., 2015).

Catalysis of Signalosome Assembly by Swapped Dimerization

Dimerization by domain swapping requires (1) a high monomer concentration and (2) conformational instability of the swapping element in the monomer (Rousseau et al., 2003). The first condition is met by the clathrin-coated pits in which multiple Dishevelled molecules are juxtaposed (Figure 7), potentially reaching a very high local concentration which could be sufficient to induce DEP dimerization. Thus, they mimic the conditions during DEP crystallization, which inexorably induced domain swapping.

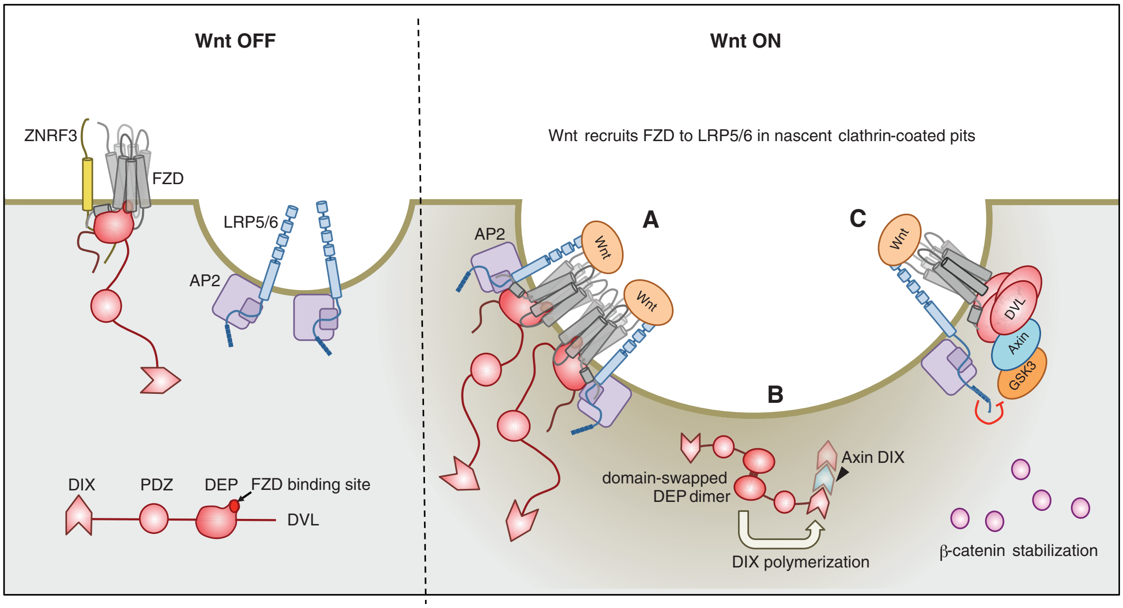

Figure 7. Model of Wnt Signalosome Assembly

Wnt OFF (left): monomeric DEP binds Frizzled to mediate ZNRF3-dependent downregulation; LRP5/6 is poised in nascent clathrin-coated pits via association with AP2μ. Wnt ON (right): (A) juxtaposition of multiple monomeric Dishevelled molecules in nascent pits triggers dimerization by DEP domain swapping, which initiates DIX-dependent polymerization and Axin co-polymerization, leading to the assembly of an active Wnt signalosome in which GSK3 is inhibited (see also Discussion). For clarity, only one complex is shown in (B), and clathrin is omitted.

The second condition may be met if Wnt binding to Frizzled were to open up its intracellular face, similarly to other agonists binding to GPCRs (Venkatakrishnan et al., 2013), thereby weakening the DEP-Frizzled contact. This is plausible, given that DEP recruitment to FZD5 decreases upon Wnt stimulation (Figure 5A) and that DEP binding depends on the base of FZD5 TM7, which is pivotal for signal transduction in Smoothened and other GPCRs (Figure S5) (Wang et al., 2013). If so, this would release the DEP finger and H1, the element being swapped during dimerization (Figure 2B), freeing it up for "conformational breathing."

Clathrin-coated pits, thus, provide both conditions necessary for triggering domain swapping of incoming DEP. Notably, domain swapping tends to be unidirectional, since the dimer, once formed, is typically more stable than the monomer (Rousseau et al., 2003). This also applies to DEP, given that its domain swapping creates a new interface, providing additional binding energy and bias toward the dimer. This bias is strongly reinforced by DEP tetramerization, which engages multiple residues that are crucial for Frizzled binding, thereby preventing re-binding of DEP to Frizzled. This mechanism of domain swapping by DEP is, therefore, an elegant structural device to achieve unidirectional regulation—a fundamental property needed for signal transduction. Owing to its unidirectionality, domain swapping drives β-sheet-dependent aggregation in neurogenerative disease, but the DEP domain provides a rare example where this mechanism controls a physiologically relevant process (Rousseau et al., 2003).

Maturation of Wnt Signalosomes

As mentioned, the boost in local concentration of Dishevelled by tetramerization could enable it to homo-polymerize and to hetero-polymerize with Axin, thereby recruiting Axin to clathrin-coated pits and into the proximity of the cytoplasmic LRP5/6 tail (Figure 7) whose phosphorylated PPPSPxS motifs constitute high-affinity Axin docking sites (MacDonald et al., 2009). LRP6 phosphorylation depends on polymerized Dishevelled and is imparted by CK1γ (Bilic et al., 2007), which may reside in nascent pits owing to a conserved YxxΦ motif (Y_{322}DWI) in its unstructured N terminus. Once phosphorylated, PPPSPxS binds directly to the catalytic cleft of GSK3 to block its activity (Stamos et al., 2014), which allows β-catenin to accumulate and to co-activate transcription in the nucleus.

Thus, Dishevelled-Axin heteropolymers remain tethered to the plasma membrane via multivalent interactions between AP2 and the various components of these Wnt signalosomes until they eventually detach (Hagemann et al., 2014), possibly as a result of phosphorylation of the relevant AP2-binding motifs, as in other examples of AP2 cargoes (Kirchhausen et al., 2014; Owen et al., 2004). This gives rise to cytoplasmic puncta (e.g., Figure 1B), which may continue to signal by sequestering Axin and antagonizing its activity in re-assembling degradasomes (Fiedler et al., 2011). However, it is also conceivable that the cytoplasmic signalosomes are no longer active in transducing a Wnt signal, being merely the remnants of the pit-associated active signalosomes.

Roles of Arrestin or G Proteins in Signalosome Assembly?

We considered the possibility that the DEP dimerization may require a Frizzled ligand that competes with DEP binding to release the DEP monomer from Frizzled. We ruled out

Dishevelled PDZ, because this domain fails to bind to Frizzled (Pan et al., 2004; Tauriello et al., 2012) (Figure 5A). β-arrestin-2 (β-arr2) was another candidate, since this Arrestin paralog binds to Frizzled in a Dishevelled-dependent way (Chen et al., 2003) and is important for Wnt signal transduction to β-catenin (Bryja et al., 2007). However, the Arrestin-binding site, as determined in rhodopsin (Kang et al., 2015), appears to overlap extensively with the DEP-binding site in FZD5 (Figures 5C and S5), implying mutually exclusive binding. β-arr2 is, thus, unlikely to catalyze signalosome assembly, but it may function subsequently during signalosome maturation, e.g., by blocking the re-binding of monomeric DEP to Frizzled, thereby reinforcing the equilibrium shift to to dimeric DEP.

G proteins could also potentially participate in DEP-dependent signalosome assembly, although their role in Wnt signal transduction remains uncertain (Dijksterhuis et al., 2014). Notably, none of the Frizzled proteins contain a DRY motif, found in TM3 of all class A GPCRs (Venkatakrishnan et al., 2013) and crucial for binding to Gs via a direct contact between its C terminus and the central arginine of this motif (Carpenter et al., 2016; Rasmussen et al., 2011). Indeed, the G protein interfaces of these two class A GPCRs are similar to one another, and their projections onto FZD5 suggest extensive overlap with its putative DEP interface (Figures 5C and S5). Again, this implies mutually exclusive binding and argues against a role of G proteins in catalyzing DEP-dependent Wnt signalosome assembly.

Cross-Linking of DIX Filaments by DEP Dimerization

The DEP-dependent dimerization of Dishevelled can provide the cross-links between DIX filaments requisite for networking them into three-dimensional signalosome structures. Therefore, it appears that the linkage of DIX polymers by DEP dimerization both catalyzes assembly of Wnt signalosomes and promotes their growth into large efficient signaling platforms with high avidity for signaling effectors. Therefore, the molecular design principle underlying Wnt signalosome assembly is beautifully parsimonious, hinging primarily on the co-operation of two domains in a single hub protein and on swapped dimerization, ensuring unidirectionality of signal transduction. Notably, the Wnt signaling pathway is one of a handful of ancient cell communication pathways found even in the most primitive animals that lack axes and tissues (Ringrose et al., 2013). The mechanistic simplicity of the Wnt signalosome may, therefore, reflect a primordial design principle.

EXPERIMENTAL PROCEDURES

Plasmids and Antibodies

The following plasmids were used: human DVL2-GFP and FLAG-Axin (Fiedler et al., 2011); FLAG-DVL2, E499G, K446M, and D460K (Mund et al., 2015); and SNAP-FZD5 (Koo et al., 2012). DEP-RFP (red fluorescent protein) was generated by subcloning DEP into DsRed. DEP and FZD5 mutants were generated by standard procedures and verified by sequencing. The following antibodies and resins were used: α-FLAG, α-tubulin, and α-GFP (Sigma); α-actin (Abcam); and α-SNAP (NE Biolabs).

Cell-Based Assays

HEK293T, HeLa, and COS-7 cells were cultured and transfected, and coIPs were conducted essentially as described previously (Mund et al., 2015). Single confocal images were acquired at identical settings with a Zeiss Confocal Microscope. For SuperTOP assays (Veeman et al., 2003), HEK293T cells were lysed 16 hr after transfection and analyzed with the Dual-Glo Luciferase Reporter Assay (Promega) according to the manufacturer's protocol. Values were normalized to Renilla luciferase and are shown as mean ± SEM relative to vector controls.

Protein Purification, Biophysics, and Crystallography

Lip-DEP$_{416–511}$ was expressed in *E. coli* BL21-CodonPlus(DE3)-RIL cells (Stratagene) and purified essentially as described previously (Fiedler et al., 2011), and the tag was removed by tobacco etch virus (TEV) protease for NMR and crystallography (Supplemental Information). SEC-MALS was performed in PBS, using a GE Superdex S-200 10/300 analytical column, and analyzed as described previously (Madrzak et al., 2015). The NMR spectroscopy is described in the Supplemental Information.

ACCESSION NUMBERS

The accession numbers for the coordinates and structure factors reported in this paper are PDB: 5LNP (mono1), 5SUZ (mono2), and 5SUY (dimer1).

SUPPLEMENTAL INFORMATION

Supplemental Information includes Supplemental Experimental Procedures, seven figures, and two tables and can be found with this article online at http://dx.doi.org/10.1016/j.molcel.2016.08.026.

AUTHOR CONTRIBUTIONS

M.V.G. and M.B. conceived and supervised the study, M.V.G. and M.R. performed most experiments, C.M.J. and T.J.R. contributed the biophysical analysis, and M.B. wrote the manuscript with input from all authors.

ACKNOWLEDGMENTS

We thank Madelon Maurice and Maike de la Roche for plasmids; Diamond Light Source for beamtime (beamlines I04 and I04-1) and assistance; Harry Powell for help with data processing; and Hugh Pelham, Richard Sear, and Steve Harrison for discussions. This work was supported by the Medical Research Council (MC_U105192713) and Cancer Research UK (C7379/A15291).

Received: May 12, 2016
Revised: July 15, 2016
Accepted: August 23, 2016
Published: September 29, 2016

REFERENCES

Axelrod, J.D., Miller, J.R., Shulman, J.M., Moon, R.T., and Perrimon, N. (1998). Differential recruitment of Dishevelled provides signaling specificity in the planar cell polarity and Wingless signaling pathways. Genes Dev. *12*, 2610–2622.

Bernatík, O., Šedová, K., Schille, C., Ganji, R.S., Červenka, I., Trantírek, L., Schambony, A., Zdráhal, Z., and Bryja, V. (2014). Functional analysis of dishevelled-3 phosphorylation identifies distinct mechanisms driven by casein kinase 1E and frizzled5. J. Biol. Chem. *289*, 23520–23533.

Bhanot, P., Fish, M., Jemison, J.A., Nusse, R., Nathans, J., and Cadigan, K.M. (1999). Frizzled and Dfrizzled-2 function as redundant receptors for Wingless during Drosophila embryonic development. Development *126*, 4175–4186.

Bienz, M. (2014). Signalosome assembly by domains undergoing dynamic head-to-tail polymerization. Trends Biochem. Sci. *39*, 487–495.

Bilic, J., Huang, Y.L., Davidson, G., Zimmermann, T., Cruciat, C.M., Bienz, M., and Niehrs, C. (2007). Wnt induces LRP6 signalosomes and promotes dishevelled-dependent LRP6 phosphorylation. Science *316*, 1619–1622.

Boutros, M., Paricio, N., Strutt, D.I., and Mlodzik, M. (1998). Dishevelled activates JNK and discriminates between JNK pathways in planar polarity and wingless signaling. Cell *94*, 109–118.

Brangwynne, C.P., Eckmann, C.R., Courson, D.S., Rybarska, A., Hoege, C., Gharakhani, J., Jülicher, F., and Hyman, A.A. (2009). Germline P granules are liquid droplets that localize by controlled dissolution/condensation. Science *324*, 1729–1732.

Bryja, V., Gradl, D., Schambony, A., Arenas, E., and Schulte, G. (2007). β-arrestin is a necessary component of Wnt/β-catenin signaling in vitro and in vivo. Proc. Natl. Acad. Sci. USA *104*, 6690–6695.

Carpenter, B., Nehmé, R., Warne, T., Leslie, A.G., and Tate, C.G. (2016). Structure of the adenosine A(2A) receptor bound to an engineered G protein. Nature *536*, 104–107.

Chen, W., ten Berge, D., Brown, J., Ahn, S., Hu, L.A., Miller, W.E., Caron, M.G., Barak, L.S., Nusse, R., and Lefkowitz, R.J. (2003). Dishevelled 2 recruits β-arrestin 2 to mediate Wnt5A-stimulated endocytosis of Frizzled 4. Science *301*, 1391–1394.

Chu, M.L., Ahn, V.E., Choi, H.J., Daniels, D.L., Nusse, R., and Weis, W.I. (2013). Structural studies of Wnts and identification of an LRP6 binding site. Structure *21*, 1235–1242.

Clevers, H., and Nusse, R. (2012). Wnt/β-catenin signaling and disease. Cell *149*, 1192–1205.

Cong, F., Schweizer, L., and Varmus, H. (2004). Wnt signals across the plasma membrane to activate the β-catenin pathway by forming oligomers containing its receptors, Frizzled and LRP. Development *131*, 5103–5115.

Consonni, S.V., Maurice, M.M., and Bos, J.L. (2014). DEP domains: structurally similar but functionally different. Nat. Rev. Mol. Cell Biol. *15*, 357–362.

Dijksterhuis, J.P., Petersen, J., and Schulte, G. (2014). WNT/Frizzled signalling: receptor-ligand selectivity with focus on FZD-G protein signalling and its physiological relevance: IUPHAR Review 3. Br. J. Pharmacol. *171*, 1195–1209.

Fiedler, M., Mendoza-Topaz, C., Rutherford, T.J., Mieszczanek, J., and Bienz, M. (2011). Dishevelled interacts with the DIX domain polymerization interface of Axin to interfere with its function in down-regulating β-catenin. Proc. Natl. Acad. Sci. USA *108*, 1937–1942.

Hagemann, A.I., Kurz, J., Kauffeld, S., Chen, Q., Reeves, P.M., Weber, S., Schindler, S., Davidson, G., Kirchhausen, T., and Scholpp, S. (2014). In vivo analysis of formation and endocytosis of the Wnt/β-catenin signaling complex in zebrafish embryos. J. Cell Sci. *127*, 3970–3982.

Hu, J., Yuan, Q., Kang, X., Qin, Y., Li, L., Ha, Y., and Wu, D. (2015). Resolution of structure of PIP5K1A reveals molecular mechanism for its regulation by dimerization and dishevelled. Nat. Commun. *6*, 8205.

Jiang, X., Charlat, O., Zamponi, R., Yang, Y., and Cong, F. (2015). Dishevelled promotes Wnt receptor degradation through recruitment of ZNRF3/RNF43 E3 ubiquitin ligases. Mol. Cell *58*, 522–533.

Kang, Y., Zhou, X.E., Gao, X., He, Y., Liu, W., Ishchenko, A., Barty, A., White, T.A., Yefanov, O., Han, G.W., et al. (2015). Crystal structure of rhodopsin bound to arrestin by femtosecond X-ray laser. Nature *523*, 561–567.

Kim, I., Pan, W., Jones, S.A., Zhang, Y., Zhuang, X., and Wu, D. (2013). Clathrin and AP2 are required for PtdIns(4,5)P2-mediated formation of LRP6 signalosomes. J. Cell Biol. *200*, 419–428.

Kirchhausen, T., Owen, D., and Harrison, S.C. (2014). Molecular structure, function, and dynamics of clathrin-mediated membrane traffic. Cold Spring Harb. Perspect. Biol. *6*, a016725.

Koo, B.K., Spit, M., Jordens, I., Low, T.Y., Stange, D.E., van de Wetering, M., van Es, J.H., Mohammed, S., Heck, A.J., Maurice, M.M., and Clevers, H. (2012). Tumour suppressor RNF43 is a stem-cell E3 ligase that induces endocytosis of Wnt receptors. Nature *488*, 665–669.

Krissinel, E., and Henrick, K. (2007). Inference of macromolecular assemblies from crystalline state. J. Mol. Biol. *372*, 774–797.

Li, P., Banjade, S., Cheng, H.C., Kim, S., Chen, B., Guo, L., Llaguno, M., Hollingsworth, J.V., King, D.S., Banani, S.F., et al. (2012). Phase transitions in the assembly of multivalent signalling proteins. Nature *483*, 336–340.

MacDonald, B.T., Tamai, K., and He, X. (2009). Wnt/β-catenin signaling: components, mechanisms, and diseases. Dev. Cell *17*, 9–26.

Madrzak, J., Fiedler, M., Johnson, C.M., Ewan, R., Knebel, A., Bienz, M., and Chin, J.W. (2015). Ubiquitination of the Dishevelled DIX domain blocks its head-to-tail polymerization. Nat. Commun. *6*, 6718.

Mund, T., Graeb, M., Mieszczanek, J., Gammons, M., Pelham, H.R., and Bienz, M. (2015). Disinhibition of the HECT E3 ubiquitin ligase WWP2 by polymerized Dishevelled. Open Biol. *5*, 150185.

Owen, D.J., Collins, B.M., and Evans, P.R. (2004). Adaptors for clathrin coats: structure and function. Annu. Rev. Cell Dev. Biol. *20*, 153–191.

Pan, W.J., Pang, S.Z., Huang, T., Guo, H.Y., Wu, D., and Li, L. (2004). Characterization of function of three domains in dishevelled-1: DEP domain is responsible for membrane translocation of dishevelled-1. Cell Res. *14*, 324–330.

Pan, W., Choi, S.C., Wang, H., Qin, Y., Volpicelli-Daley, L., Swan, L., Lucast, L., Khoo, C., Zhang, X., Li, L., et al. (2008). Wnt3a-mediated formation of phosphatidylinositol 4,5-bisphosphate regulates LRP6 phosphorylation. Science *321*, 1350–1353.

Povelones, M., Howes, R., Fish, M., and Nusse, R. (2005). Genetic evidence that Drosophila frizzled controls planar cell polarity and Armadillo signaling by a common mechanism. Genetics *171*, 1643–1654.

Punchihewa, C., Ferreira, A.M., Cassell, R., Rodrigues, P., and Fujii, N. (2009). Sequence requirement and subtype specificity in the high-affinity interaction between human frizzled and dishevelled proteins. Protein Sci. *18*, 994–1002.

Rasmussen, S.G., DeVree, B.T., Zou, Y., Kruse, A.C., Chung, K.Y., Kobilka, T.S., Thian, F.S., Chae, P.S., Pardon, E., Calinski, D., et al. (2011). Crystal structure of the β2 adrenergic receptor-Gs protein complex. Nature *477*, 549–555.

Ringrose, J.H., van den Toorn, H.W., Eitel, M., Post, H., Neerincx, P., Schierwater, B., Altelaar, A.F., and Heck, A.J. (2013). Deep proteome profiling of Trichoplax adhaerens reveals remarkable features at the origin of metazoan multicellularity. Nat. Commun. *4*, 1408.

Rothbächer, U., Laurent, M.N., Deardorff, M.A., Klein, P.S., Cho, K.W., and Fraser, S.E. (2000). Dishevelled phosphorylation, subcellular localization and multimerization regulate its role in early embryogenesis. EMBO J. *19*, 1010–1022.

Rousseau, F., Schymkowitz, J.W., and Itzhaki, L.S. (2003). The unfolding story of three-dimensional domain swapping. Structure *11*, 243–251.

Schulte, G., and Bryja, V. (2007). The Frizzled family of unconventional G-protein-coupled receptors. Trends Pharmacol. Sci. *28*, 518–525.

Schwarz-Romond, T., Merrifield, C., Nichols, B.J., and Bienz, M. (2005). The Wnt signalling effector Dishevelled forms dynamic protein assemblies rather than stable associations with cytoplasmic vesicles. J. Cell Sci. *118*, 5269–5277.

Schwarz-Romond, T., Fiedler, M., Shibata, N., Butler, P.J., Kikuchi, A., Higuchi, Y., and Bienz, M. (2007). The DIX domain of Dishevelled confers Wnt signaling by dynamic polymerization. Nat. Struct. Mol. Biol. *14*, 484–492.

Sear, R.P. (2008). Phase separation of equilibrium polymers of proteins in living cells. Faraday Discuss. *139*, 21–34.

Stamos, J.L., Chu, M.L., Enos, M.D., Shah, N., and Weis, W.I. (2014). Structural basis of GSK-3 inhibition by N-terminal phosphorylation and by the Wnt receptor LRP6. eLife *3*, e01998.

Strutt, D., Madder, D., Chaudhary, V., and Artymiuk, P.J. (2012). Structure-function dissection of the frizzled receptor in Drosophila melanogaster suggests different mechanisms of action in planar polarity and canonical Wnt signaling. Genetics *192*, 1295–1313.

Tauriello, D.V., Jordens, I., Kirchner, K., Slootstra, J.W., Kruitwagen, T., Bouwman, B.A., Noutsou, M., Rüdiger, S.G., Schwamborn, K., Schambony, A., and Maurice, M.M. (2012). Wnt/β-catenin signaling requires interaction of the Dishevelled DEP domain and C terminus with a discontinuous motif in Frizzled. Proc. Natl. Acad. Sci. USA *109*, E812–E820.

Umbhauer, M., Djiane, A., Goisset, C., Penzo-Méndez, A., Riou, J.F., Boucaut, J.C., and Shi, D.L. (2000). The C-terminal cytoplasmic Lys-thr-X-X-X-Trp motif in frizzled receptors mediates Wnt/β-catenin signalling. EMBO J. *19*, 4944–4954.

Veeman, M.T., Slusarski, D.C., Kaykas, A., Louie, S.H., and Moon, R.T. (2003). Zebrafish prickle, a modulator of noncanonical Wnt/Fz signaling, regulates gastrulation movements. Curr. Biol. *13*, 680–685.

Venkatakrishnan, A.J., Deupi, X., Lebon, G., Tate, C.G., Schertler, G.F., and Babu, M.M. (2013). Molecular signatures of G-protein-coupled receptors. Nature *494*, 185–194.

Wang, C., Wu, H., Katritch, V., Han, G.W., Huang, X.P., Liu, W., Siu, F.Y., Roth, B.L., Cherezov, V., and Stevens, R.C. (2013). Structure of the human smoothened receptor bound to an antitumour agent. Nature *497*, 338–343.

Wilkins, D.K., Grimshaw, S.B., Receveur, V., Dobson, C.M., Jones, J.A., and Smith, L.J. (1999). Hydrodynamic radii of native and denatured proteins measured by pulse field gradient NMR techniques. Biochemistry *38*, 16424–16431.

Wong, H.C., Mao, J., Nguyen, J.T., Srinivas, S., Zhang, W., Liu, B., Li, L., Wu, D., and Zheng, J. (2000). Structural basis of the recognition of the dishevelled DEP domain in the Wnt signaling pathway. Nat. Struct. Biol. *7*, 1178–1184.

Wong, H.C., Bourdelas, A., Krauss, A., Lee, H.J., Shao, Y., Wu, D., Mlodzik, M., Shi, D.L., and Zheng, J. (2003). Direct binding of the PDZ domain of Dishevelled to a conserved internal sequence in the C-terminal region of Frizzled. Mol. Cell *12*, 1251–1260.

Wu, H., and Fuxreiter, M. (2016). The structure and dynamics of higher-order assemblies: amyloids, signalosomes, and granules. Cell *165*, 1055–1066.

Wu, J., Jenny, A., Mirkovic, I., and Mlodzik, M. (2008). Frizzled-Dishevelled signaling specificity outcome can be modulated by Diego in Drosophila. Mech. Dev. *125*, 30–42.

Yu, A., Rual, J.F., Tamai, K., Harada, Y., Vidal, M., He, X., and Kirchhausen, T. (2007). Association of Dishevelled with the clathrin AP-2 adaptor is required for Frizzled endocytosis and planar cell polarity signaling. Dev. Cell *12*, 129–141.

Yu, A., Xing, Y., Harrison, S.C., and Kirchhausen, T. (2010). Structural analysis of the interaction between Dishevelled2 and clathrin AP-2 adaptor, a critical step in noncanonical Wnt signaling. Structure *18*, 1311–1320.

Molecular Cell, Volume *64*

Supplemental Information

Wnt Signalosome Assembly by DEP Domain Swapping of Dishevelled

Melissa V. Gammons, Miha Renko, Christopher M. Johnson, Trevor J. Rutherford, and Mariann Bienz

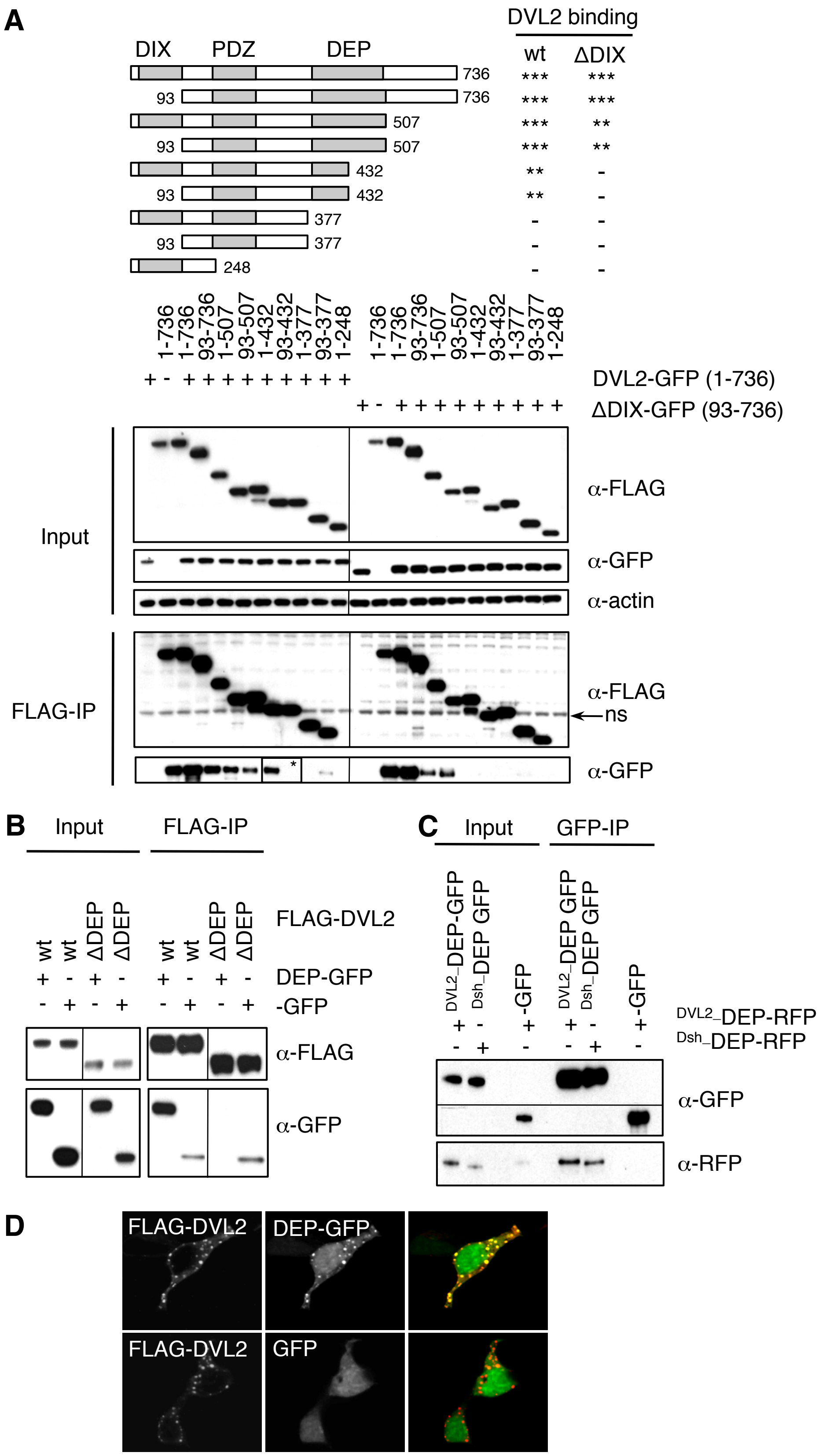

Fig. S1 (related to main Fig. 1)

DEP-dependent self-association of DVL2. (A-C) Western blots of coIPs from HEK293T cell lysates, 24 hours after transfection; (A) truncation mapping of DVL2 self-association reveals dependence on DEP, as summarized above; ns, non-specific band. (B-C) Mutual binding between DEP414-507-GFP and FLAG-DVL2 but not ΔDEP, or between DEP414-507-RFP and DEPDVL2-GFP or DEPDsh-GFP. (D) Confocal images of HEK293T cells co-expressing FLAG-DVL2 (red) and DEP414-507-GFP (green), revealing recruitment of DEP into DVL2 puncta; scale bar, 10μm. Lines indicate where blots have been spliced together, *lanes reversed due to misload.

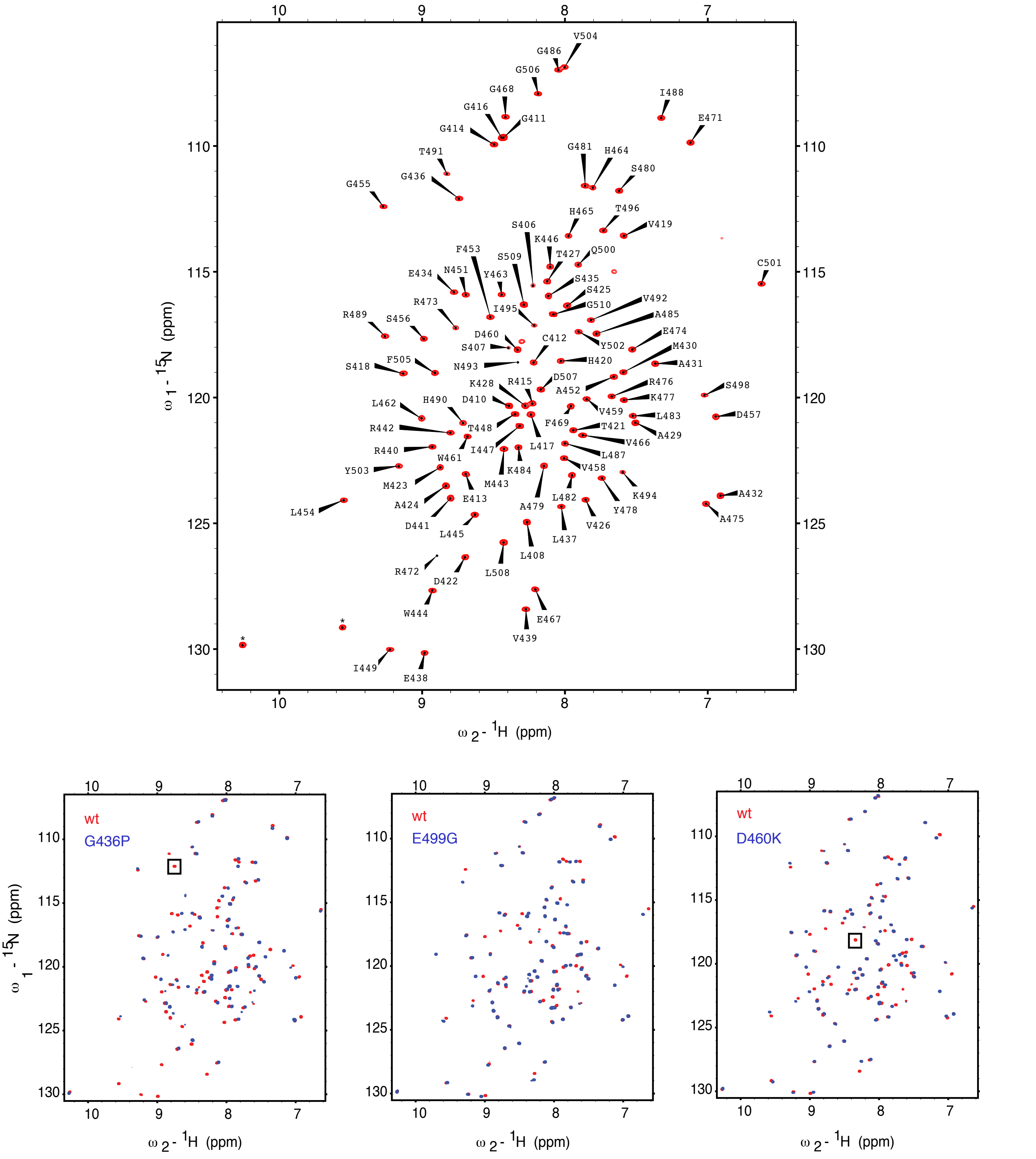

Fig. S2 (related to main Fig. 3)

BEST-TROSY spectra of wt and mutant DVL2 DEP. Top, 15N-1H correlation spectrum of purified 15N-13C-DEP416-511 monomer, with residues labelled according to backbone amide resonance assignments (except for A450, K470, N493, F497 and E499, which could not be assigned; asterisks indicate side chains); underneath, overlays of BEST-TROSY spectra of purified wt 15N-DEP416-511 (red) and mutants (blue) as indicated in panels, with mutated residues boxed (where assigned). The well-dispersed peaks of even intensity of the dimerization mutants indicate normal folding and absence of aggregation. The overall peak positioning for the wt spectrum is conserved in each of the mutants, and chemical shift mapping indicated that the only significant changes are in the vicinity of the mutated residue, although even these are relatively minor. We thus infer that the normal fold of the DEP monomer is preserved in each of the three mutants.

A

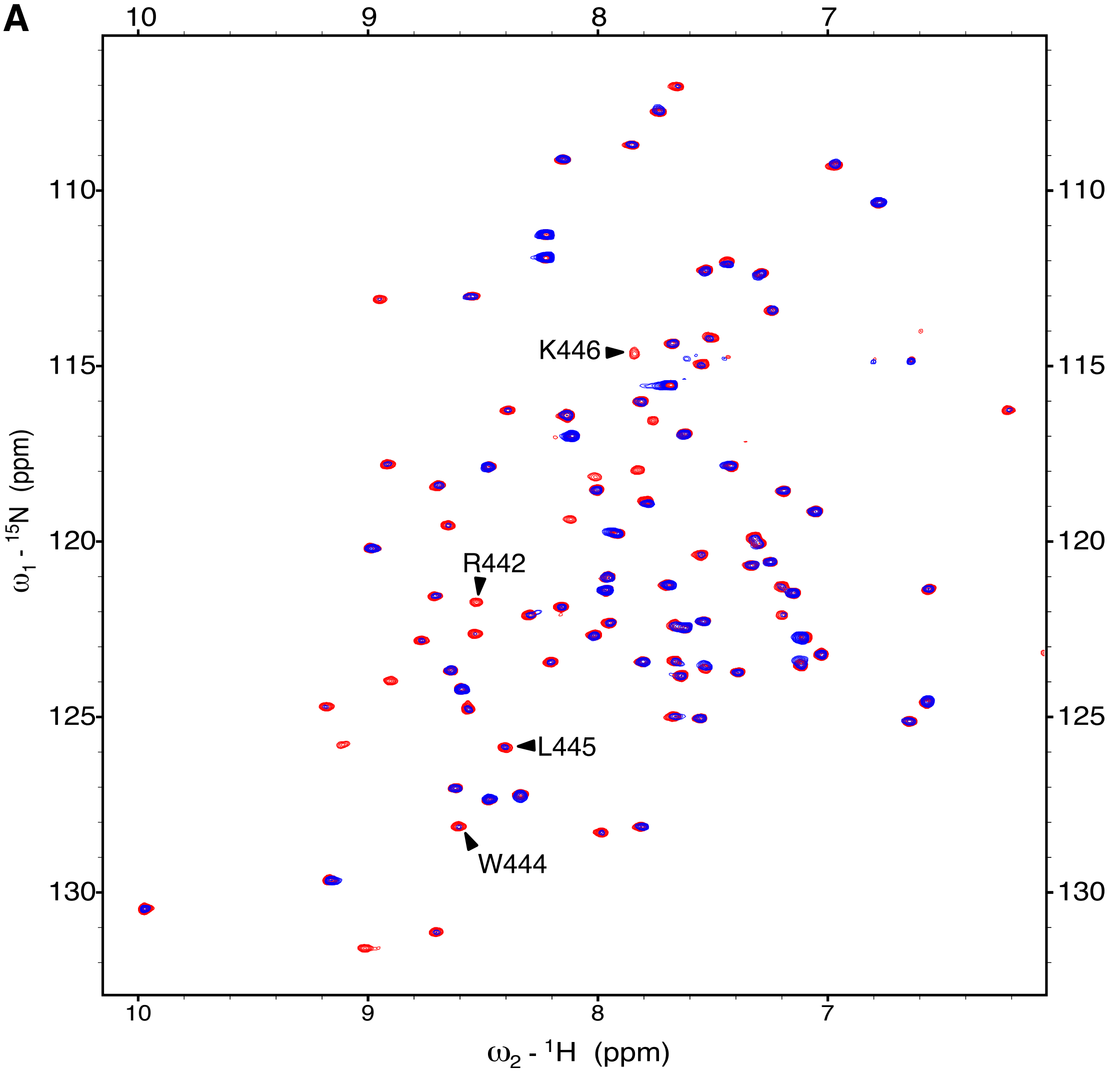

B

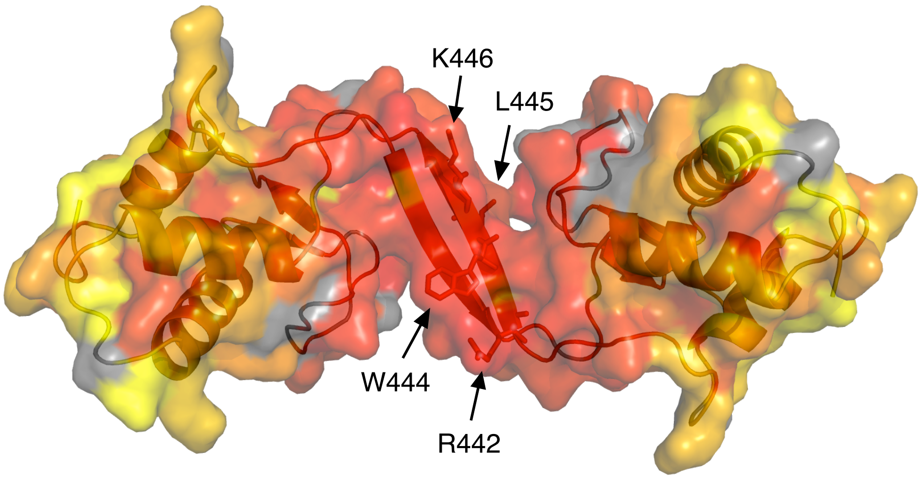

Fig. S3 (related to main Fig. 3)

BEST-TROSY spectra of monomeric and dimeric DEP. (A) Overlay of 15N-1H correlation spectra of 100 µM purified 15N-DEP416-511 monomer (red) and 15N-DEP416-511 dimer (blue); maximal line broadenings in the dimer (relative to the monomer) are observed for DEP finger residues (labeled), consistent with their radical conformational change from loop (in the monomer) to β1β2 sheet (in the dimer; see also main Fig. 2B). (B) Heat-map of relative line broadenings (dimer/monomer), ranging from 32.5% (yellow) to 77.4% reduction of absolute peak height (red), revealing maximal line broadenings in the β1β2 link region; grey, prolines and unassigned residues (see also Fig. S2).

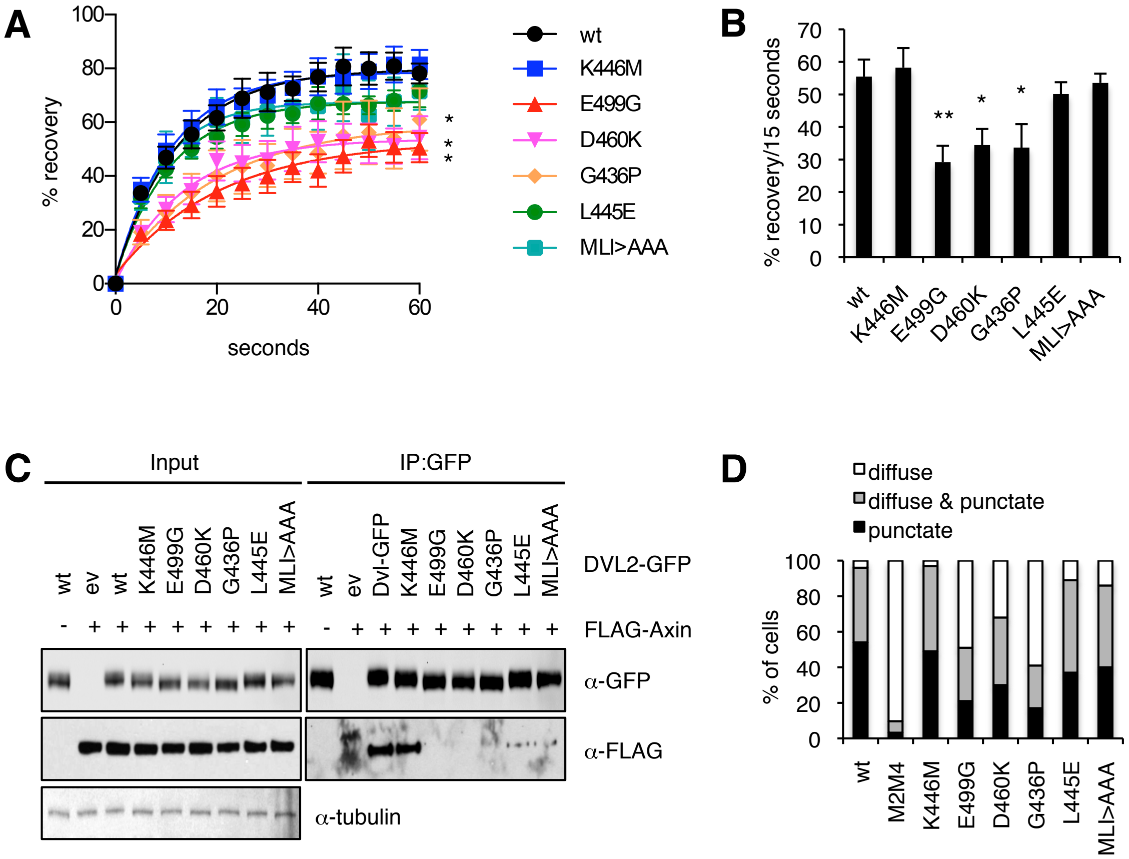

Fig. S4 (related to main Fig. 4)

Additional characterization of DEP mutants. (A, B) FRAP analysis of COS-7 cells expressing wt or mutant DVL2-GFP, as described (Metcalfe et al., 2010); >5 puncta were bleached per individual cell (>5 cells analyzed per mutant), and the fluorescence intensity was recorded every 5 seconds; (A) mean traces, with SEM indicated; (B) Percent recovery at 15 seconds; statistical significance, ** $p<0.01$, * $p<0.05$ (One-way ANOVA, Dunnetts post-hoc). (C) CoIP assays in transfected HEK293T cells, co-expressing DVL2-GFP and FLAG-Axin, as in main Fig. 1A. (D) Quantitation of puncta formation in HeLa cells expressing wt or mutant DVL2-GFP, as in main Fig. 2B (see also main Fig. 4D).

A

```
                     TM1                                    ICL1         TM2

FZD5        229 ADERTFATFWIGLWSVLCFISTSTTVATFLI  DMER-FRYP    ERPIIFLSACYLCV-SLGFLVRL
Smo         224 EAEHQDMHSYIAAFGAVTGLCTLFTLATFVA  DWRNSNRYP    AVILFYVNACFFVG-SIGWLAQF
Rhodopsin    34 PWQFSMLAAYMFLLIVLGFPINFLTLYVTVQH  KKL-RT   PLNYILLNLAVADLFMVLGGFTSTLYTSLH
beta2AR      31  VWVVGMGIVMSLIVLAIVFGNVLVITAIAK   FERL-QT  VTNYFITSLACADLVMGLAVVPFGAAHILMK
A2AR          7      SVYITVELAIAVLAILGNVLVCWAVWL    NSNL-QN  VTNYFVVSLAAADIAVGVLAIPFAITISTGF

                ECL1                              TM3

FZD5        290 VVGHASVACSREHNHIHYETTGP       ALCTIVFLLVYFFGMASSIWWVILSLTWFLAAGMKW
Smo         264 MDGARREIVCRADGTMRLGEPTSNET    LSCVIIFVIVYYALMAGVVWFVVLTYAWHTSFKALG
Rhodopsin   101 GYFVFG                       PTGCNLEGFFATLGGEIALWSLVVLAIERYVVVC
beta2AR      98 MWTFG                        NFWCEFWTSIDVLCVTASIETLCVIAVDRYFAITS
A2AR         71 CAA                          CHGCLFIACFVLVLTQSSIFSLLAIAIDRYIAIR

                ICL2                 TM4                ECL2

FZD5        349 GNE-AIAGYAQYF          HLAAWLIPSVKSITALAL  SSVDGDPVAGICYVGNQN
Smo         348 TTYQPLSGKTSYF          HLLTWSLPFVLTVAILAV  AQVDGDSVSGICFVGYKN
Rhodopsin   141    KPMSNF-RFG   ENHAIMGVAFTWVMALACAAPPLA  GWSRYIPEGLQCSCGIDYYTLKPEVN
beta2AR     138      PFKYQ-SLL  TKNKARVIILMVWIVSGLTSFLPIQM  HWYRATHQEAINCYANETCCDFFT
A2AR        108     IPLRYN-GLVT  GTRAKGIIAICWVLSFAIGLTPML  GWNNCGQPKEGKNHSQGCGEGQVACLFEDVVP

                TM5                                     ICL3

FZD5        397 LNSLRGFVLGP-LVLYLLVGTLFLLAGFVSLFRIRSV    IKQGG------------------TKT
Smo         397 YRYRAGFVLAP-IGLVLIVGGYFLIRGVMTLFSIKSN    HPGLLS-----------------EKAA
Rhodopsin   200 NESFVIYMFVVHFTIPMIIIFFCYGQLVFTVKEAAAQ    -QQ-------------------ES-A
beta2AR     196 NQAYAIASSIVSFYVPLVIMVFVYSRVFQEAKRQLQKIDKSE GRFHVQNLSQVEQDGRTGHGLRRSSKF
A2AR        174 MNYMVYFNFFACVLVPLLLMLGVYLRIFLAARRQLKQM    ESQPLPG------------ERARSTLQ

                TM6                                                    ECL3

FZD5        441 DKLEKLMIRIGIFTLLLYTVPASIVVACYLYEQHYRESWEAALTCACPGHD  TGQPRAKPE
Smo         443 SKINETMLRLGIFGFLAFGFVLITFSCHFYDFFNQAEWERSFRDYVLCQA  NVTIGLPTKQPIPDCEIKNRPS
Rhodopsin   242 TTQKAEKEVTRMVIIMVIAFLICWVPYASVAFYIFTH                  QGSNFGP
beta2AR     265   CLKEHKALKTLGIIMGTFTLCWLPFFIVNIVHVIQ                  DNLI
A2AR        227      KEVHAAKSLAIIVGLFALCWLPLHIINCFTFF                   CPDCSHAP

                TM7                          H8

FZD5        500      YWVLMLKYFMCLVVGITSGVWI  WS  GKTVESWRRF
Smo         515      LLVEKINLFAMFGTGIAMSTWV  WT  KATLLIWRRT
Rhodopsin   286   IFMTIPAFFAKS-AAIYNPVIYIMMN     KQFRNCMLTTIC
beta2AR     304 RKEVYILLNWIGYV-NSGFNPLIYCR    S  PDFRIAFQEL
A2AR        267  LWLMYLAIVLSHT-NSVVNPFIYAYR   I  REFRQTFRKIIRSHVL
```

B

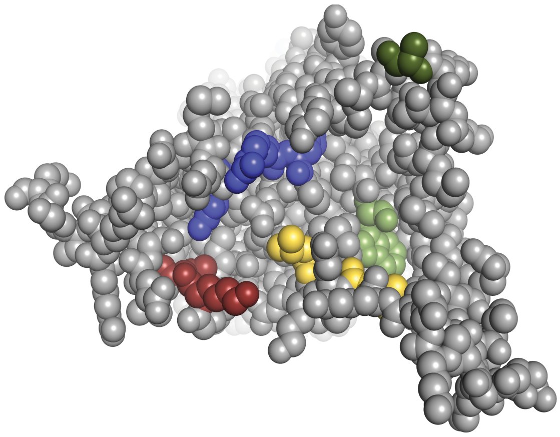

Fig. S5 (related to main Fig. 5)

Structural alignments of GPCRs. (A) Sequence alignments of human GPCRs mentioned in this study, based on structural projections generated by gpcrdb.org, but adopting the limits of TM segments (TM1-7) and α-helix 8 (H8) of Smoothened (Smo) and FZD5 from Fig. S1 (4JKV; Wang et al., 2013), of the adenosine A2A (A2AR) and β-adrenergic (beta2AR) receptors from Fig. S5 (Carpenter et al., 2016; see also 3SN6; Rasmussen et al., 2011), and of Rhodopsin from 4ZWJ (Kang et al., 2015). Sequence similarity was taken into account for alignments of intracellular loop sequences (ICL1-3) within and, where possible, across the two classes (although the cross-class alignments are arbitrary as these loops are largely unstructured, exhibiting little if any sequence conservation); extracellular segments (ECL1-3) were left-aligned, for simplicity. Blue, X50 residues in Ballesteros & Weinstein numbering as identified by gpcrdb.org, except for 2.50 whose topological equivalent in Smoothened is F274 (Y278 in FZD5), according to Fig. S1 (Wang et al., 2013) and our own structural cross-class alignments of TM2 segments. Residues required (red, orange) or dispensable (turquoise) for DEP recruitment to FZD5 are colored as in main Fig. 5C (red, expression level and modification comparable to wt FZD5; orange, abnormal expression, i.e. low level, or unmodified, or proteolyzed). Residues contacting Gs (green) or Arrestin (purple) were also colored; for Gs, see Fig. 3 & S5 (Carpenter et al., 2016); for Arrestin, see cross-linking data in Fig. 5 (Kang et al., 2015); ICL1 also contributes to Arrestin binding (Kang et al., 2015) although there are no cross-linking data for this loop. (B) Variant on-view of DEP binding to intracellular face of FZD5, as defined by residues required for DEP recruitment (red in A); rainbow colors as in main Fig. 5D. This model, based on the structure of Smoothened in complex with cyclopamine (4O9R; Weierstall et al., 2014), presents a somewhat more 'open' face compared to that shown in main Fig. 5D (based on 4JKV, the structure of Smoothened in complex with LY2940680; Wang et al., 2013), fully exposing W340 (light green), the topological equivalent of the arginine in DRY motif at the apex of the Gs binding pocket which, in class A GPCRs, engages in a crucial contact with the C-terminus of Gs (Carpenter et al., 2016; Rasmussen et al., 2011). Both models were generated by Phyre2.

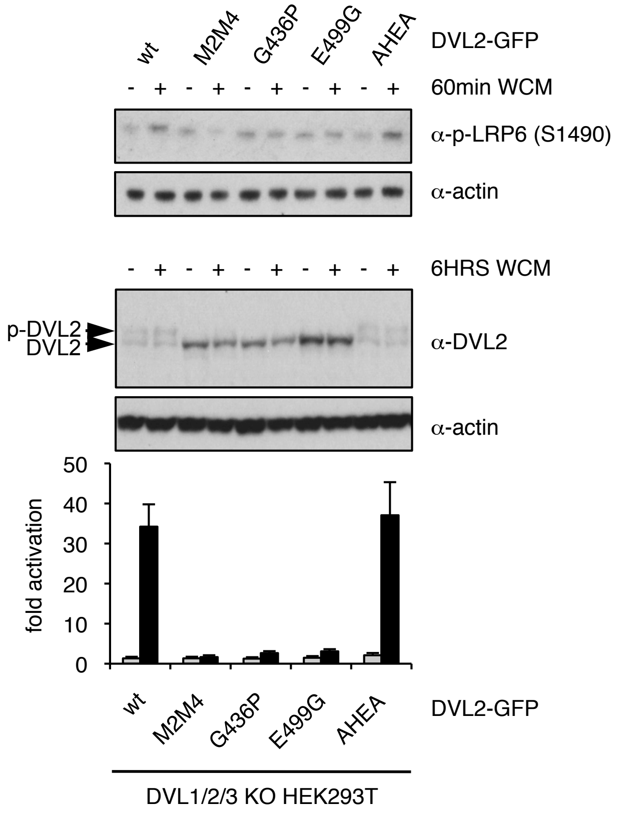

Fig. S6 (related to main Fig. 7)

Requirement for DEP-dependent dimerization during Wnt signaling. Complementation assays of wt or mutant DVL2-GFP expressed at physiological levels with a pBABEpuro retroviral vector in HEK293T cells lacking endogenous DVL1-3. (A) SuperTOP assays monitoring signaling activities of complementing transgenes (with corresponding Western blot above), as in main Fig. 1C. (B) GSK3-dependent phosphorylation of LRP6 (Zeng et al., 2005), as a more direct read-out of Dishevelled signaling activity (Bilic et al., 2007). Notably, these signaling activities of DVL2-GFP are strictly Wnt-dependent as they are detectable only after stimulation with Wnt3a-conditioned media (WCM, for 6 hours or 60 minutes, as indicated), but they do not depend on binding to AP2m since mutation of the m2-binding YHEL motif (located downstream of the DEP domain) to AHEA (Yu et al., 2010) has no detectable effect on function in these complementation assays.

Note: Our characterisation of the complementation assay is now in press (Gammons, M., Rutherford, T.J., Steinhart, Z., Angers, S., and Bienz, M. Essential role of the Dishevelled DEP domain in a Wnt-dependent human cell-based complementation assay. J. Cell Sci. 2016)

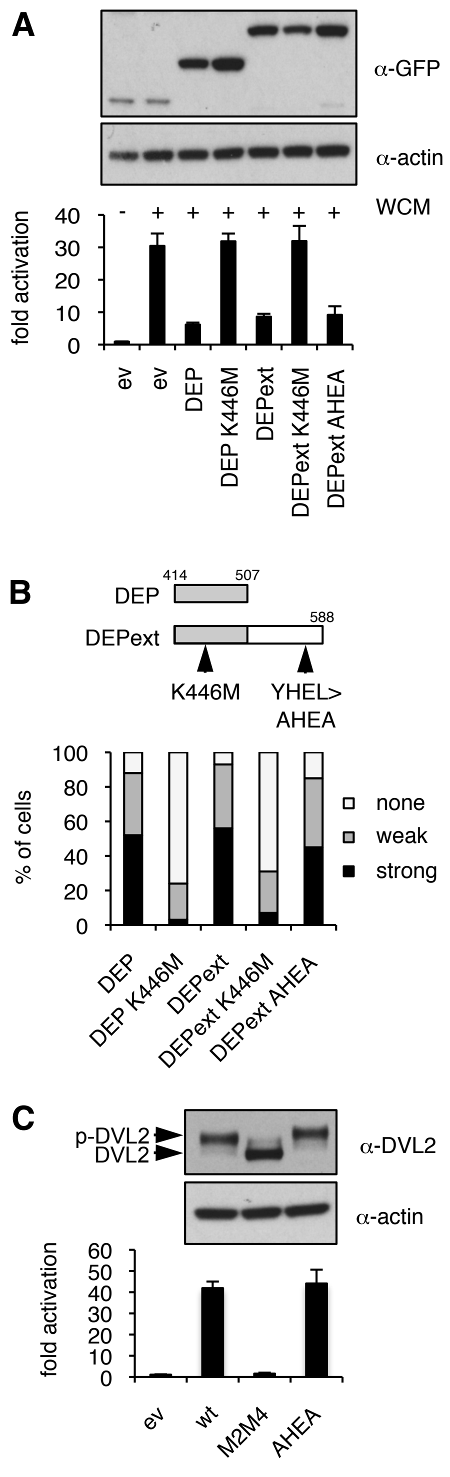

Fig. S7 (related to main Fig. 7)

Additional functional testing of the DVL2 YHEL motif. (A) SuperTOP assays in transiently transfected HEK293T cells, as in main Fig. 1C (with corresponding Western blot above). (B) SuperTOP assays, monitoring blocking of Wnt signal transduction by *endogenous* Dishevelled, as in main Fig. 6A (*above*, corresponding Western blot). (C) PM recruitments assays in HEK293T cells co-transfected with SNAP-FZD5 plus DEP-GFP, or plus an extended DEP domain including YHEL (DEPext-GFP; amino acids 414-588; Yu et al., 2010), as in main Fig. 6B. None of these assays uncovered a requirement for DVL2 binding to AP2m (blocked by the AHEA mutation; Yu et al., 2010) which appeared dispensable for Wnt-independent (A) and Wnt-dependent signaling (B), and also for recruitment to FZD5.

mutation	coIP	recruitment to DVL2 puncta	DVL2 signalling activity
S418A	Y	Y	+
T421A	Y	Y	+++
D422A	Y	Y	nt
S425A	Y	Y	+++
K428A	Y	reduced	nt
A432R	Y	Y	nt
E434R	Y	Y	nt
S435A	Y	Y	+
E438K	Y	Y	nt
R440H	Y	Y	nt
R442H	Y	Y	nt
R442W	Y	Y	nt
W444A	Y	Y	nt
K446M	Y	Y	++
K446E	Y	Y	++
T449R	Y	Y	nt
P450A	Y	Y	+++
E471A	Y	Y	nt
E471K	Y	Y	nt
R473E	Y	Y	nt
A475V	Y	Y	+
K477E	Y	Y	nt
Y478A	Y	Y	nt
S480A	Y	Y	++
I488N	Y	Y	+
T491R	Y	Y	nt
K494A	Y	Y	++
S498A	Y	Y	nt
Q500A	Y	Y	nt
C501R	Y	Y	+++
Y502F	Y	Y	+++

Table S1 (related to main Fig. 2)

Mutation screening of DEP surface residues. Effects of individual amino acid substitutions of DEP surface residues on DEP or DVL2 function in transfected HEK293T cells; first column, coIP of mutant DEP-GFP with wt DEP-RFP (as in Fig. S1C); second column, recruitment of mutant DEP-GFP to wt FLAG-Dvl2 puncta (as in Fig. S1D); third column, signaling activity of full-length Dvl2-GFP bearing DEP mutation; +++, wt; ++, >50% of wt; +, <50% of wt; nt, not tested.

PDB ID	5SUY	5LNP	5SUZ
Condition	0.1 M MES, pH=6.5<br>0.1 M NaCl<br>0.1 M Li_2SO_4<br>23 % PEG400	0.1 M MES, pH=6.5<br>0.1 M NaCl<br>0.1 M Li_2SO_4<br>23 % PEG400	0.1 M MES, pH=6.5<br>0.2 M $CaCl_2$<br>23 % PEG350 MME
Resolution range	40.5 - 1.89 (1.96 - 1.89)	42.0 - 1.99 (2.06 - 1.99)	32.7 - 1.84 (1.91 - 1.84)
Space group	$P2_1$	$P2_1$	C2
Unit cell	61.67, 60.17, 66.13,<br>90, 117.62, 90	61.58, 60.18, 66.29<br>90, 117.71, 90	117.79, 59.60, 30.32<br>90, 93.64, 90
Total reflections	236719 (3397)	200819	239437 (23533)
Unique reflections	34407 (3423)	29398 (2884)	17446 (1733)
Multiplicity	6.9 (6.9)	6.8 (6.6)	13.7 (13.6)
Complicity (%)	99.2 (98.3)	99.0 (98.6)	92.0 (96.0)
Mean I/sigma(I)	13.1 (1.8)	17.3 (2.5)	16.1 (0.98)
R-merge	0.085 (0.963)	0.064 (0.636)	0.107 (2.416)
R-meas	0.093 (1.042)	0.070 (0.690)	0.111 (2.511)
CC1/2	0.999 (0.767)	0.999 (0.889)	1 (0.937)
Twin fraction (%)	20.2	46.7	n/a
Twin operator	-h, -k, h+l	-h, -k, h+l	n/a
Reflections used in refinement	34407 (3423)	29398 (2884)	16251 (1215)
Reflections used for R-free	1837 (191)	1559 (150)	852 (79)
R-work	0.185	0.194	0.217
R-free	0.217	0.246	0.263
Number of non-hydrogen atoms:	3147	3031	1601
-macromolecules	3015	2993	1519
-ligands	15	5	n/a
Protein residues	378	377	190
RMS (bonds)	0.016	0.016	0.012
RMS (angles)	1.59	1.75	1.57
Ramachandran favored (%)	98	96	99
Ramachandran allowed (%)	2.1	3	0.53
Ramachandran outliers (%)	0	0.54	0.53
Rotomer outliers (%)	0.31	5.1	4.9
Clashscore	1.16	2.69	1.98
Average B-factor	33.05	37.09	39.83
-macromolecules	32.87	37.12	39.71
-ligands	55.20	47.43	n/a
-solvent	34.86	32.18	41.94

Statistics for the highest-resolution shell are shown in parentheses.

Table S2 (related to main Fig. 2)

Crystallography data collection and refinement statistics

SUPPLEMENTAL EXPERIMENTAL PROCEDURES

X-ray structure determination and refinement

Crystallization was done with concentrated DEP protein (20 mg/ml) after removal of tag by TEV protease (leaving two serine residues at the N-terminal end of DEP), as described (Stock et al., 2005); the initial screen involved ~1500 different crystallization conditions in 100 nl drops in a 96-well sitting-drop format. Crystals emerged under multiple conditions after growing for 30 days at 19ºC by the vapor diffusion method, and were directly flash-frozen in liquid nitrogen. X-ray diffraction data were collected at 100 K with a Dectris Pilatus 6M detector using the Diamond Light Source beamlines I04 and I04-1, from crystals grown in 0.1 M MES, pH 6.5, 0.2 M $CaCl_2$, 23 % PEG350 MME (C2 crystal form) or 0.1 M MES, pH 6.5, 0.1 M NaCl, 0.1 M Li_2SO4 and 22 % PEG400 ($P2_1$ crystal form). The C-centered monoclinic structure was determined by single anomalous dispersion using selenomethionine-labelled crystals. Determination of the heavy atom substructure and initial CA-model building were done using SHELXD (Sheldrick, 2010). The structure was built with ArpWarp (Langer et al., 2013) and manually edited subsequently with COOT (Emsley et al., 2010). The primitive monoclinic crystals were merohedrally twinned, with variable twinning fraction (20-45%). These structures were determined by molecular replacement with Phaser (McCoy et al., 2007), included in the CCP4 suite (Winn et al., 2011), using the C-centered monoclinic structure as a search model. All structures were refined using Refmac (Murshudov et al., 2011), and structural images were drawn with PyMol.

NMR spectroscopy

NMR spectra were acquired on Bruker Avance-III spectrometers operating at 600 or 800 MHz ^{1}H frequency, and equipped with cryogenic inverse 5 mm probes. Backbone resonance frequencies were obtained for 350 μM ^{13}C-^{15}N-labelled protein at 298 K, using unmodified Bruker pulse programs for HNCACB, CBCA(CO)NH, HN(CA)CO and HNCO. {^{1}H,^{15}N}-BEST-TROSY spectra (Favier and Brutscher, 2011) were acquired with 128 complex and 1024 points in t_1 and t_2, respectively, and 32 transients per t_1 point, for purified ^{15}N-labelled DEP monomer (at 298 K for **Fig. S2**, or at 283K for **Fig. S3**), or dimer (at 278 K; **Fig. S3**). In each case, 100 μM protein in aqueous phosphate buffer at physiological ionic strength (pH 6.7) was used. BEST-TROSY spectra were also obtained for ^{15}N-labelled DEP monomer or dimer after incubation with 300 μM of a 12-mer peptide from FZD5 (GKTLESWRRFTS) (Tauriello et al., 2012) which spans H8, but no chemical shift perturbation or line broadening was observed. Likewise, there was no observable chemical shift perturbation nor line broadening if the same 12-mer was ^{15}N-labelled as a Lip-tagged peptide (separated from the N-terminal tag by the linker ENLYFQS encoding a TEV cleavage site) and incubated at 100 μM with 300 μM purified DEP monomer. This indicates a low affinity between this H8 peptide and the DEP domain (K_d>1 mM), likely because the peptide contributes only a small number of residues to the whole interface between FZD5 and DEP (see main **Fig. 5C, D**), and maybe also because it lacks helical conformation which, in the equivalent peptide from FZD1, depends on association with micelles (Gayen et al., 2013).

SUPPLEMENTAL REFERENCES

Emsley, P., Lohkamp, B., Scott, W.G., and Cowtan, K. (2010). Features and development of Coot. Acta Crystallogr D Biol Crystallogr *66*, 486-501.

Favier, A., and Brutscher, B. (2011). Recovering lost magnetization: polarization enhancement in biomolecular NMR. J Biomol NMR *49*, 9-15.

Gayen, S., Li, Q., Kim, Y.M., and Kang, C. (2013). Structure of the C-terminal region of the Frizzled receptor 1 in detergent micelles. Molecules *18*, 8579-8590.

Langer, G.G., Hazledine, S., Wiegels, T., Carolan, C., and Lamzin, V.S. (2013). Visual automated macromolecular model building. Acta Crystallogr D Biol Crystallogr *69*, 635-641.

McCoy, A.J., Grosse-Kunstleve, R.W., Adams, P.D., Winn, M.D., Storoni, L.C., and Read, R.J. (2007). Phaser crystallographic software. J Appl Crystallogr *40*, 658-674.

Metcalfe, C., Mendoza-Topaz, C., Mieszczanek, J., and Bienz, M. (2010). Stability elements in the LRP6 cytoplasmic tail confer efficient signalling upon DIX-dependent polymerization. J Cell Sci *123*, 1588-1599.

Murshudov, G.N., Skubak, P., Lebedev, A.A., Pannu, N.S., Steiner, R.A., Nicholls, R.A., Winn, M.D., Long, F., and Vagin, A.A. (2011). REFMAC5 for the refinement of macromolecular crystal structures. Acta Crystallogr D Biol Crystallogr *67*, 355-367.

Sheldrick, G.M. (2010). Experimental phasing with SHELXC/D/E: combining chain tracing with density modification. Acta Crystallogr D Biol Crystallogr *66*, 479-485.

Stock, D., Perisic, O., and Lowe, J. (2005). Robotic nanolitre protein crystallisation at the MRC Laboratory of Molecular Biology. Prog Biophys Mol Biol *88*, 311-327.

Weierstall, U., James, D., Wang, C., White, T.A., Wang, D., Liu, W., Spence, J.C., Bruce Doak, R., Nelson, G., Fromme, P.*, et al.* (2014). Lipidic cubic phase injector facilitates membrane protein serial femtosecond crystallography. Nat Commun *5*, 3309.

Winn, M.D., Ballard, C.C., Cowtan, K.D., Dodson, E.J., Emsley, P., Evans, P.R., Keegan, R.M., Krissinel, E.B., Leslie, A.G., McCoy, A.*, et al.* (2011). Overview of the CCP4 suite and current developments. Acta Crystallogr D Biol Crystallogr *67*, 235-242.

Zeng, X., Tamai, K., Doble, B., Li, S., Huang, H., Habas, R., Okamura, H., Woodgett, J., and He, X. (2005). A dual-kinase mechanism for Wnt co-receptor phosphorylation and activation. Nature *438*, 873-877.
